# Supplementary material for: Mutant p53 variants differentially impact replication initiation and activate cGAS-STING to affect immune checkpoint inhibition
Source: Commun Biol. 2025 Nov 5;8:1522. doi: 10.1038/s42003-025-09050-3 (PMC12589595; doi:10.1038/s42003-025-09050-3)
Supplement: Supplementary file 2 — Supplementary Information [file 42003_2025_9050_MOESM2_ESM.pdf]

Supplementary Information

Mutant p53 variants differentially impact replication initiation and activate cGAS-STING to affect immune checkpoint inhibition

Kang Liu, Lidija A. Wilhelms Garan, Fang-Tsyr Lin, and Weei-Chin Lin

Corresponding author:

Weei-Chin Lin

Email: weeichil@bcm.edu

Supplementary information contains:

Supplementary Figures S1-12

Supplementary Table S1

Supplementary References

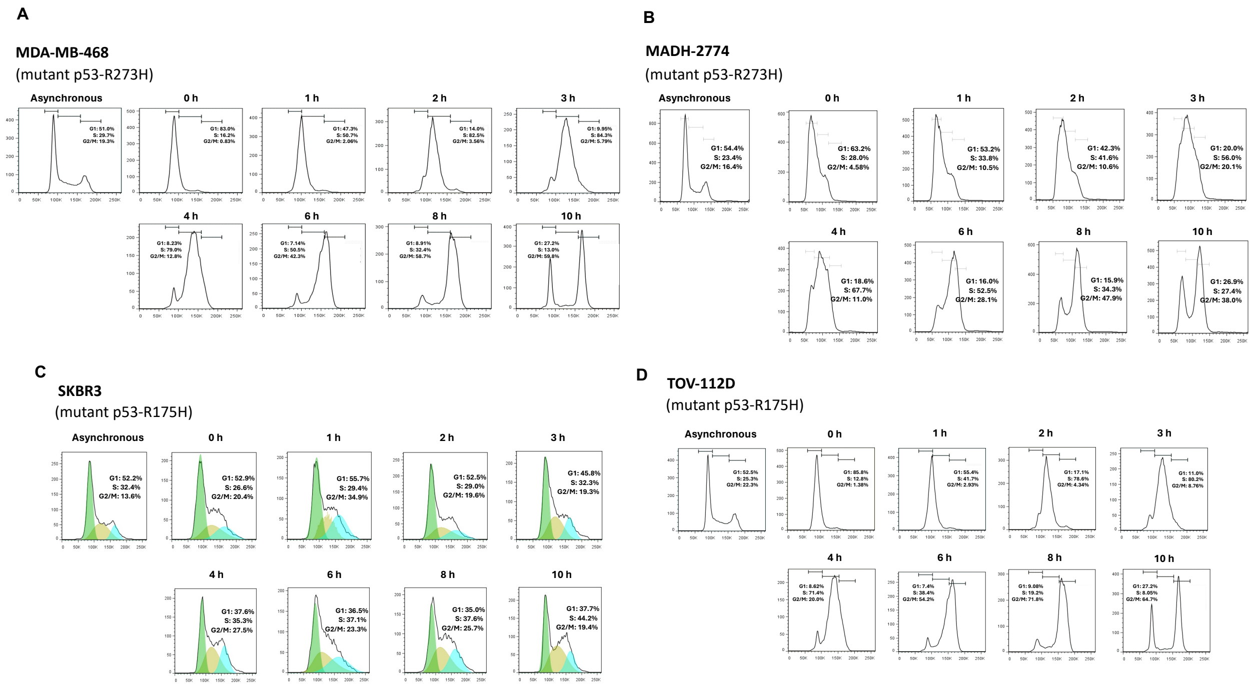

Fig. S1. Cell cycle analysis of various mutant p53-harboring cancer cells after double thymidine block and release. Related to Fig. 1.

The breast cancer cell line MDA-MB-468 (A) and ovarian cancer cell line MDAH-2774 (B), both harboring mutp53-R273H, as well as the breast cancer cell line SKBR3 (C) and ovarian cancer cell line TOV-112D (D), which harbor mutp53-R175H, were synchronized at the G1/S border using the double thymidine block procedure, as described in the Materials and Methods. After washing twice with PBS, cells were incubated in fresh medium containing 10% FBS and then were fixed at the indicated time points. DNA was stained with propidium iodide followed by flow cytometry analysis. At least 10,000 cells per sample were analyzed. The bars at the top of the graphs indicate gates that define, from left to right, the G1, S, and G2/M subpopulations.

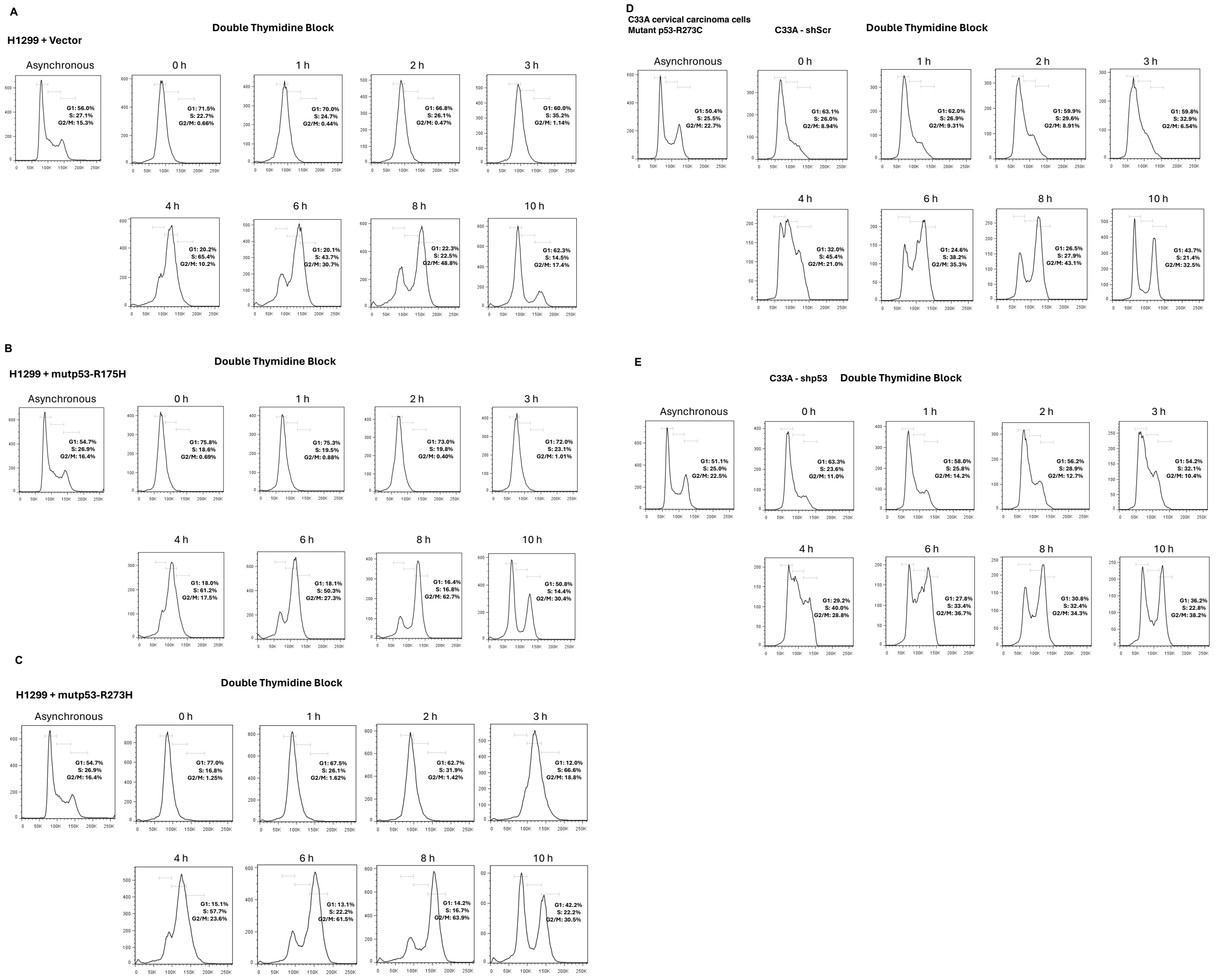

Fig. S2. Flow cytometry profile of synchronization and cell cycle progression after double thymidine block. Related to Fig. 2.

H1299 cells expressing a control vector (A), mutp53-R175H (B), or mutp53-R273H (C) were synchronized by the double thymidine block procedure. After release, cells were cultured in fresh medium containing 10% FBS, and then fixed at the indicated time points. DNA was stained with propidium iodide, followed by flow cytometry analysis. The same experimental procedures were performed in C33A cells stably expressing either shScr (D) or mutant p53 shRNA (E).

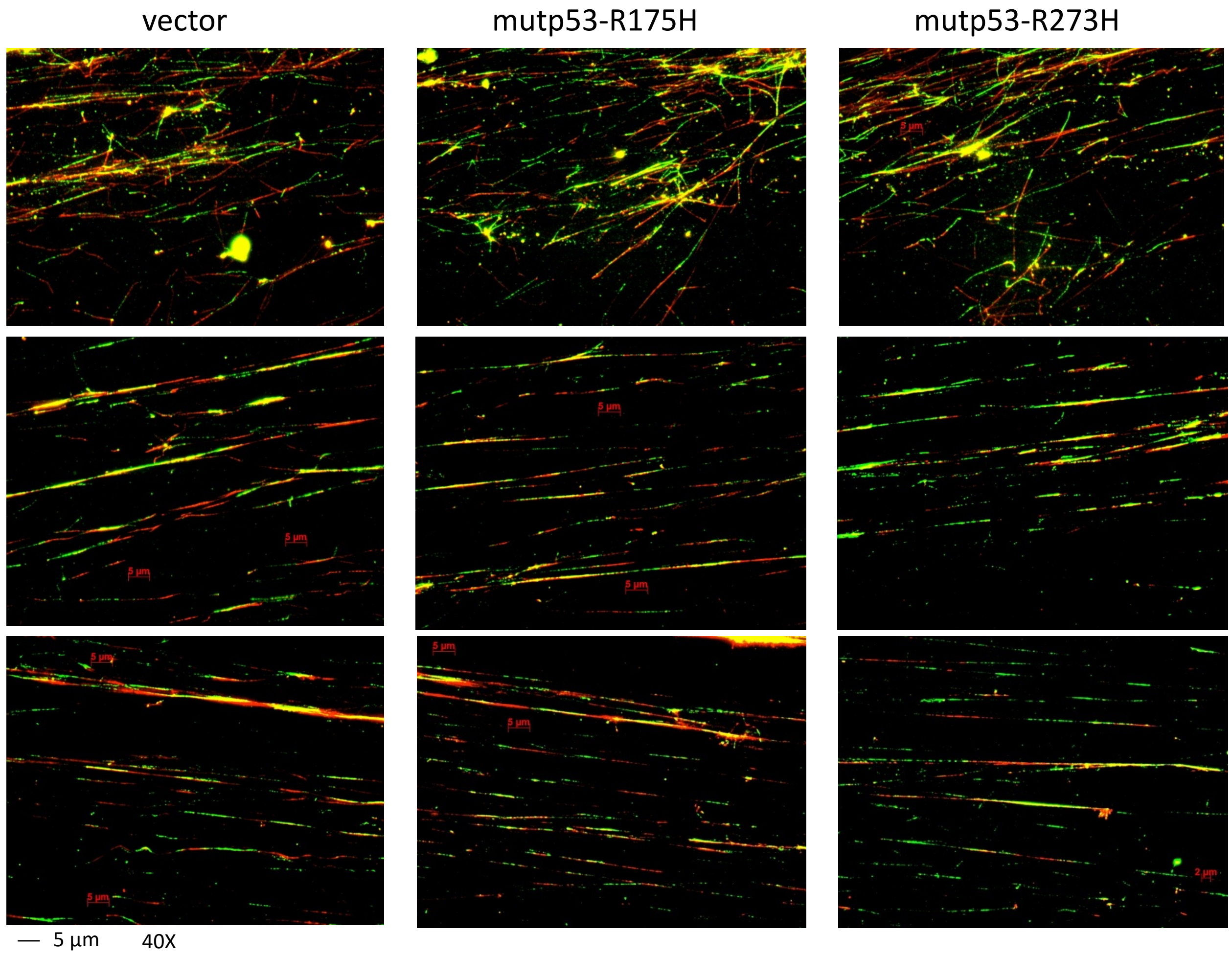

Fig. S3. Contact mutant p53 enhances the initiation of DNA replication in the late S phase.

Related to Fig. 3.

Pictures shown are representative images (at 40 X magnifications) of DNA fiber assay described in Figure 3.

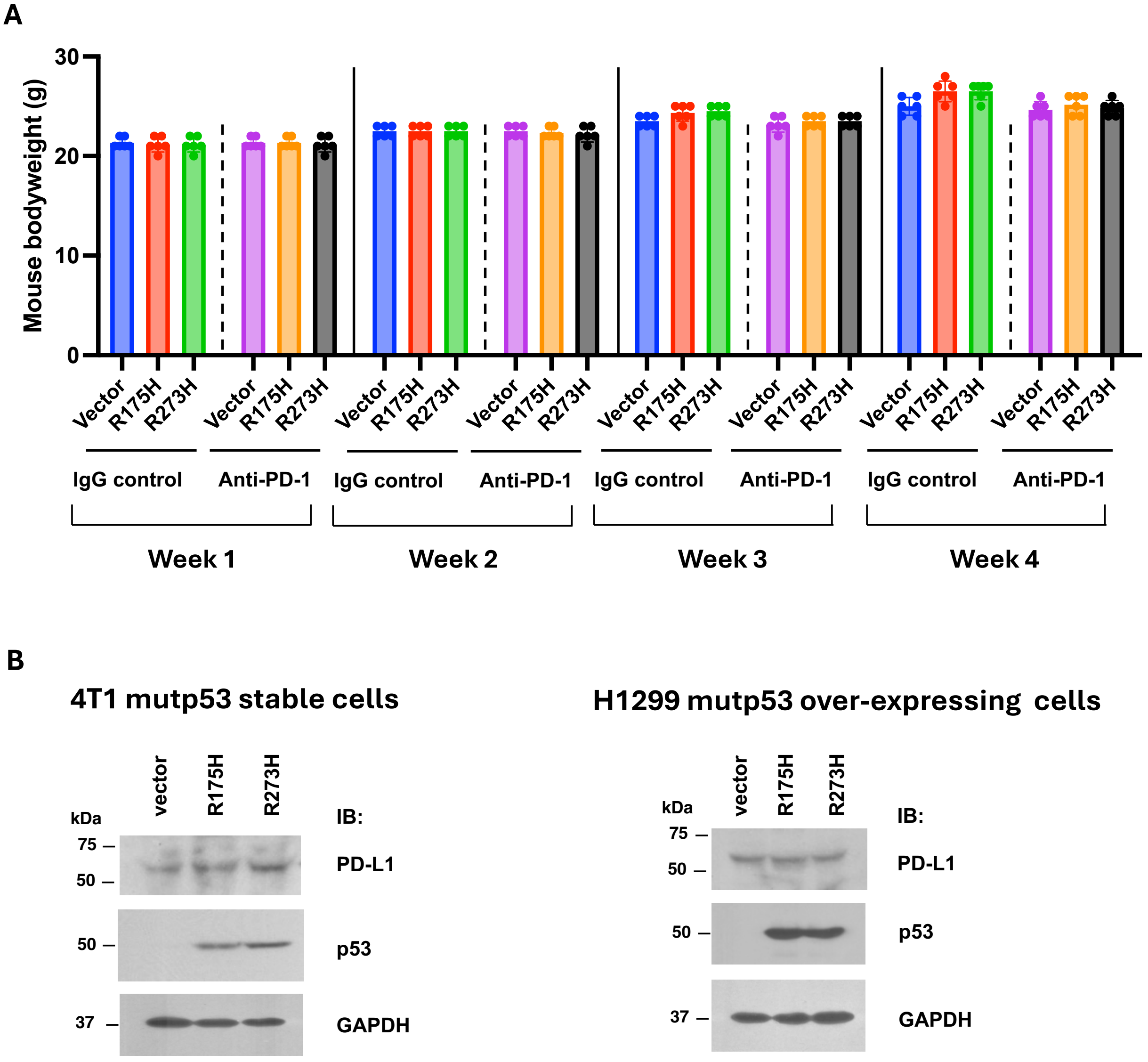

Fig. S4. Mutant p53 does not alter PD-L1 expression and also does not affect the body weight of mice injected with cancer cells harboring mutp53 after anti-PD-1 treatment. Related to Fig. 8.

(A) Body weight of mice injected with 4T1 stable cell lines harboring an empty vector, mutp53-R273H or mutp53-R175H during IgG control or anti-PD-1 treatment. (B) Western blot analysis of PD-L1 in 4T1 cells stably expressing mutp53 or in H1299 cells transiently expressing mutp53.

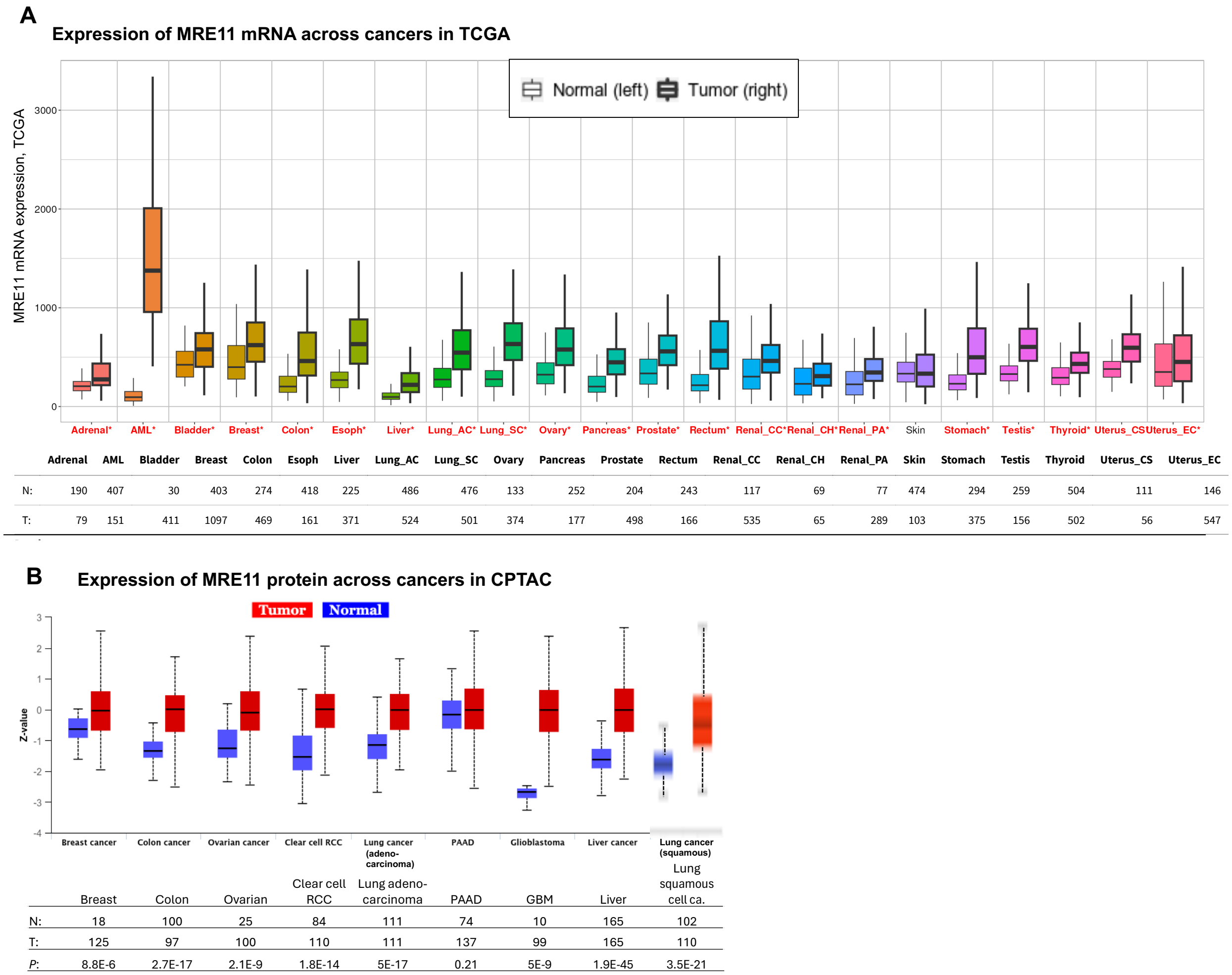

Fig. S5. *MRE11* mRNA and protein are overexpressed in many types of cancer. Related to Fig. 9.

(A) *MRE11* mRNA expression in TCGA datasets. Expression of *MRE11* is significantly elevated in most types of cancer compared to normal tissues. Tumor samples are denoted with bolded lines. The bottom panels show the numbers of normal (N) and tumor (T) samples. Significance is calculated by Mann–Whitney U test. Red* indicates $P < 0.05$. Data adapted using TNMPlot [1].

(B) MRE11 protein expression in CPTAC datasets. Sample numbers and P values are shown in the bottom panel. RCC: renal cell carcinoma; PAAD: pancreatic adenocarcinoma; GBM: glioblastoma multiforme. Data adapted using UALCAN [2].

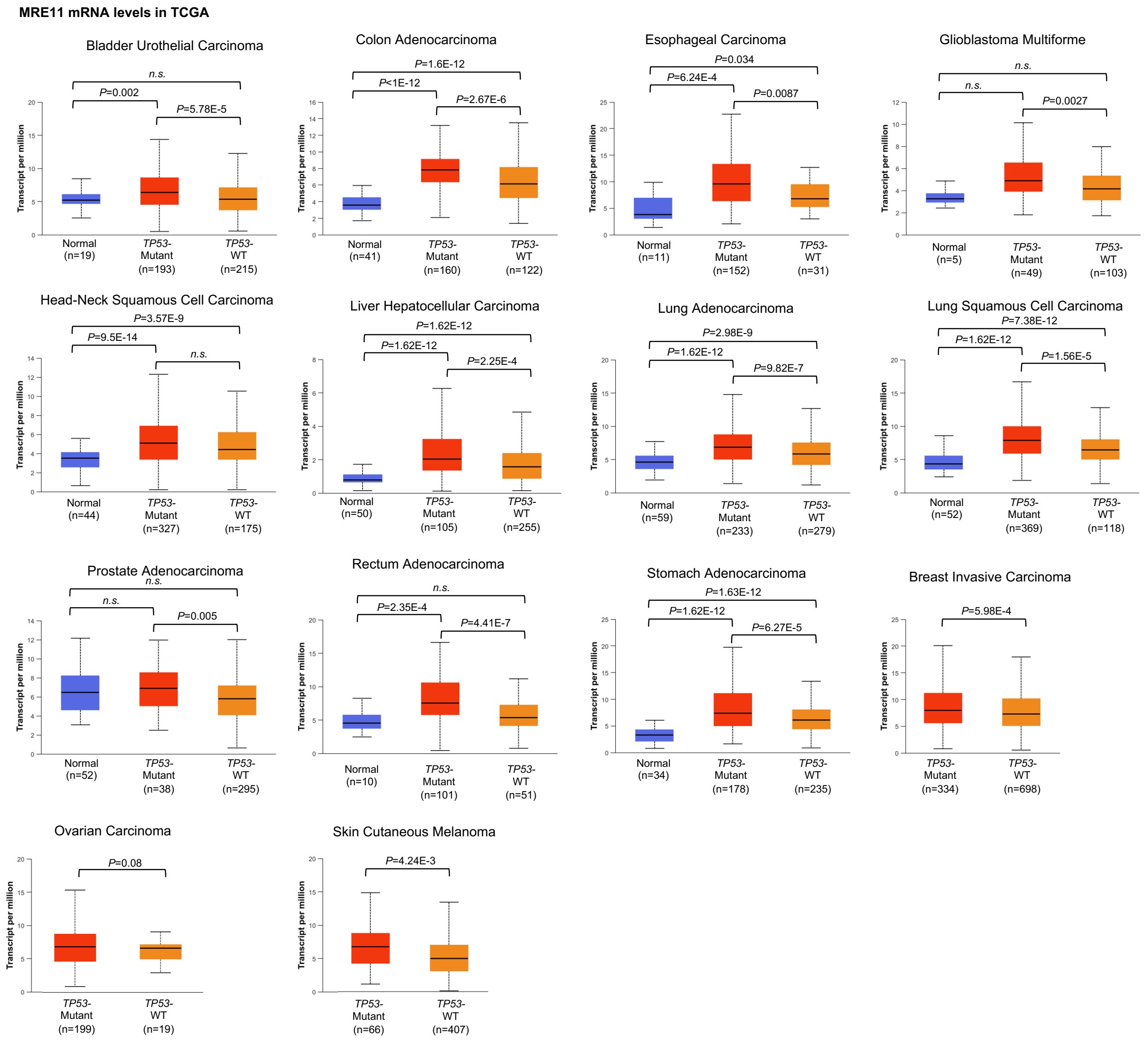

Fig. S6. MRE11 is expressed at higher levels in cancers harboring mutp53 compared to those harboring wild-type p53. Related to Fig. 9.

Expression of MRE11 in normal tissues or various types of tumors harboring wild-type or mutant p53 in TCGA datasets. P-values were calculated using a two-tailed *t*-test.

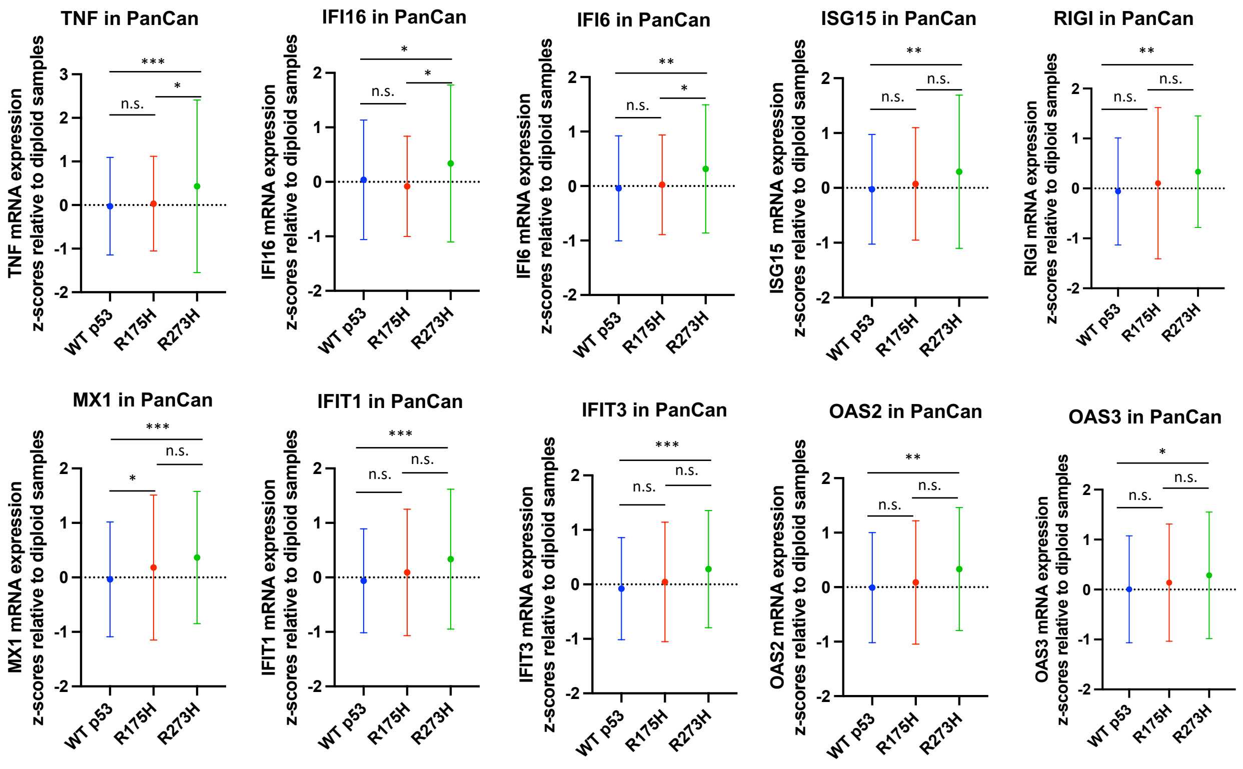

Fig. S7. Tumors harboring mutp53-R273H express higher levels of cGAS-STING target genes compared to those harboring wild-type *TP53* or mutp53-R175H. Related to Fig. 10A. mRNA expression of cGAS-STING target genes in tumors harboring either wild-type (WT) *TP53*, mutp53-R175H or mutp53-R273H in a combined TCGA Pan-Cancer database, which contains 10967 samples from 32 studies compiled by cBioPortal. Shown are mean RNA-seq z-scores (relative to diploid samples) ± SD. N = 6604 (WT), 129 (R175H) and 88 (R273H), respectively. To avoid confounding effects from different *TP53* mutations, tumors containing an additional allele of *TP53* mutation were excluded. *$P < 0.05$, **$P < 0.01$, ***$P < 0.001$, ns, not significant (two-tailed t test).

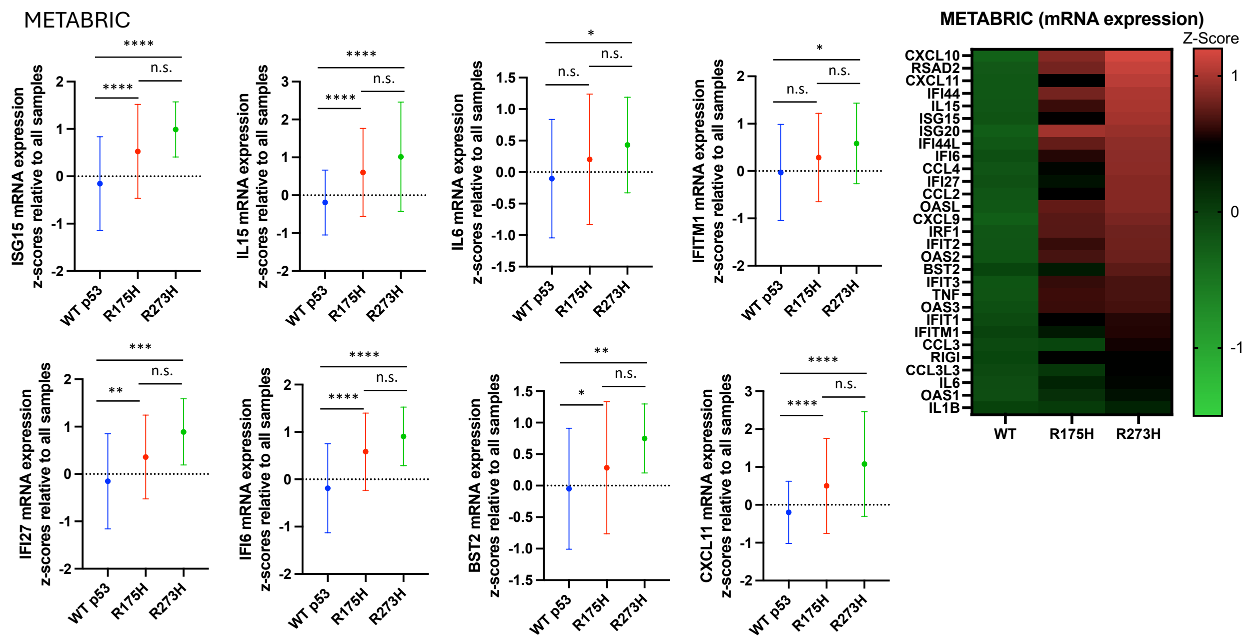

Fig. S8. Breast cancers harboring mutp53-R273H express higher levels of cGAS-STING target genes compared to those harboring wild-type *TP53* or mutp53-R175H. Related to Fig. 10A.

mRNA expression of cGAS-STING target genes in tumors harboring either wild-type (WT) *TP53*, mutp53-R175H or mutp53-R273H in METABRIC breast cancer dataset [3], which contains 2509 samples. Shown are mean z-scores (mRNA expression relative to all samples) ± SD. N = 1207 (WT), 34 (R175H) and 12 (R273H), respectively. To avoid confounding effects from different *TP53* mutations, tumors containing an additional allele of *TP53* mutation were excluded. **P* < 0.05, ***P* < 0.01, ****P* < 0.001, *****P* < 0.0001, ns, not significant (two-tailed *t* test). Right panel: Heatmap showing mean mRNA expression levels.

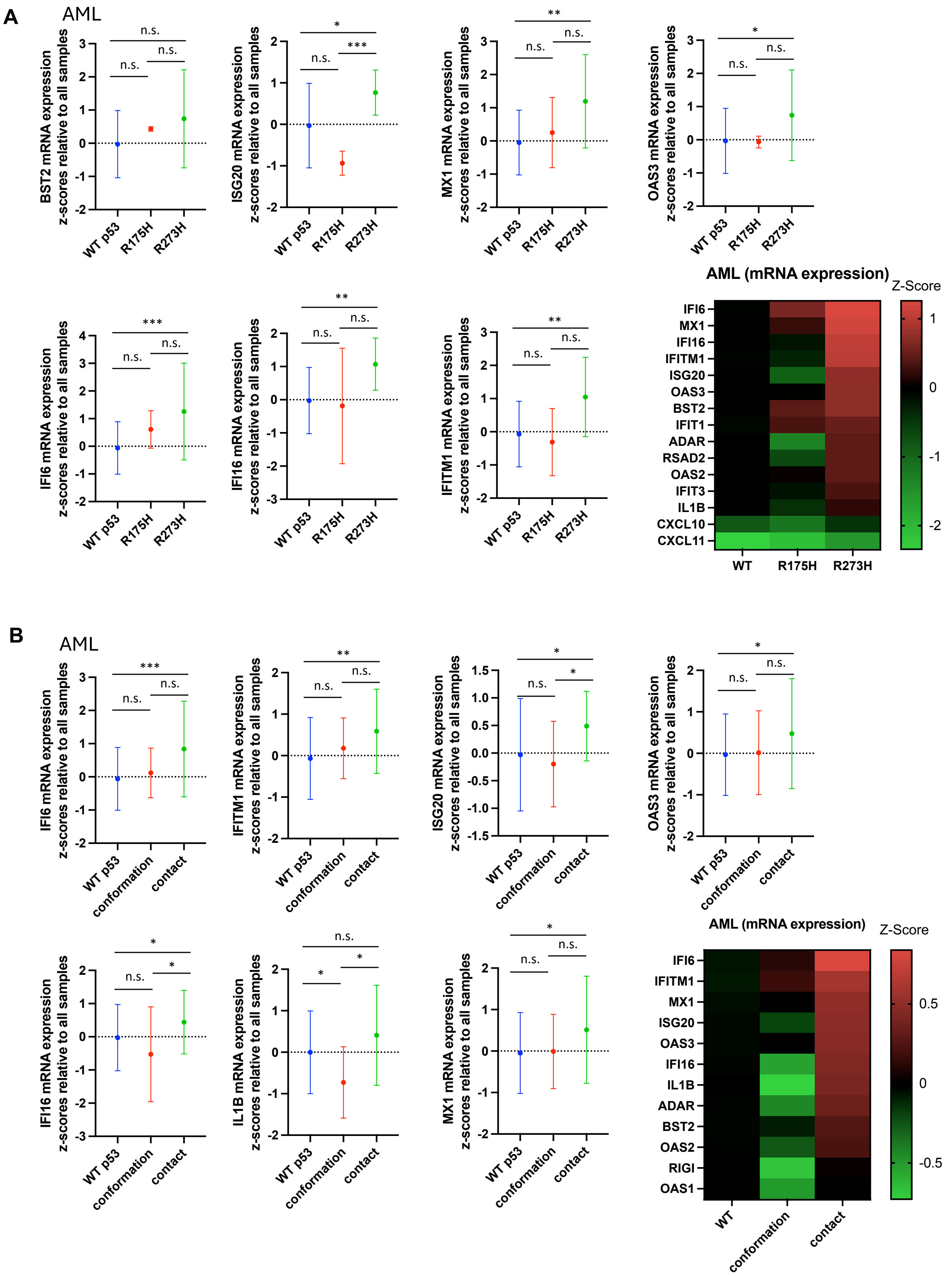
A
AML
BST2 mRNA expression z-scores relative to all samples
ISG20 mRNA expression z-scores relative to all samples
MX1 mRNA expression z-scores relative to all samples
OAS3 mRNA expression z-scores relative to all samples
IFI6 mRNA expression z-scores relative to all samples
IFI16 mRNA expression z-scores relative to all samples
IFITM1 mRNA expression z-scores relative to all samples
WT p53
R175H
R273H
n.s.
*
**
***
AML (mRNA expression)
Z-Score
IFI6
MX1
IFI16
IFITM1
ISG20
OAS3
BST2
IFIT1
ADAR
RSAD2
OAS2
IFIT3
IL1B
CXCL10
CXCL11
WT
R175H
R273H
B
AML
IFI6 mRNA expression z-scores relative to all samples
IFITM1 mRNA expression z-scores relative to all samples
ISG20 mRNA expression z-scores relative to all samples
OAS3 mRNA expression z-scores relative to all samples
IFI16 mRNA expression z-scores relative to all samples
IL1B mRNA expression z-scores relative to all samples
MX1 mRNA expression z-scores relative to all samples
WT p53
conformation
contact
AML (mRNA expression)
Z-Score
IFI6
IFITM1
MX1
ISG20
OAS3
IFI16
IL1B
ADAR
BST2
OAS2
RIGI
OAS1
WT
conformation
contact

Fig. S9. AML harboring mutp53-R273H or another contact mutp53 express higher levels of cGAS-STING target genes compared to those harboring wild-type *TP53*, mutp53-R175H or another conformational mutp53. Related to Fig. 10A.

(A) mRNA expression of cGAS-STING target genes in tumors harboring either wild-type (WT) *TP53*, mutp53-R175H or mutp53-R273H in an AML dataset [4], which contains 942 samples. (B) The analysis was expanded to encompass all contact or conformational mutp53 variants. Shown are mean z-scores (mRNA expression relative to all samples) ± SD. N = 821 (WT), 6 (R175H), 7 (R273H), 15 (conformation), and 23 (contact), respectively. *$P < 0.05$, **$P < 0.01$, ***$P < 0.001$, ns, not significant (two-tailed t test). Right bottom panel: Heatmap showing mean mRNA expression levels.

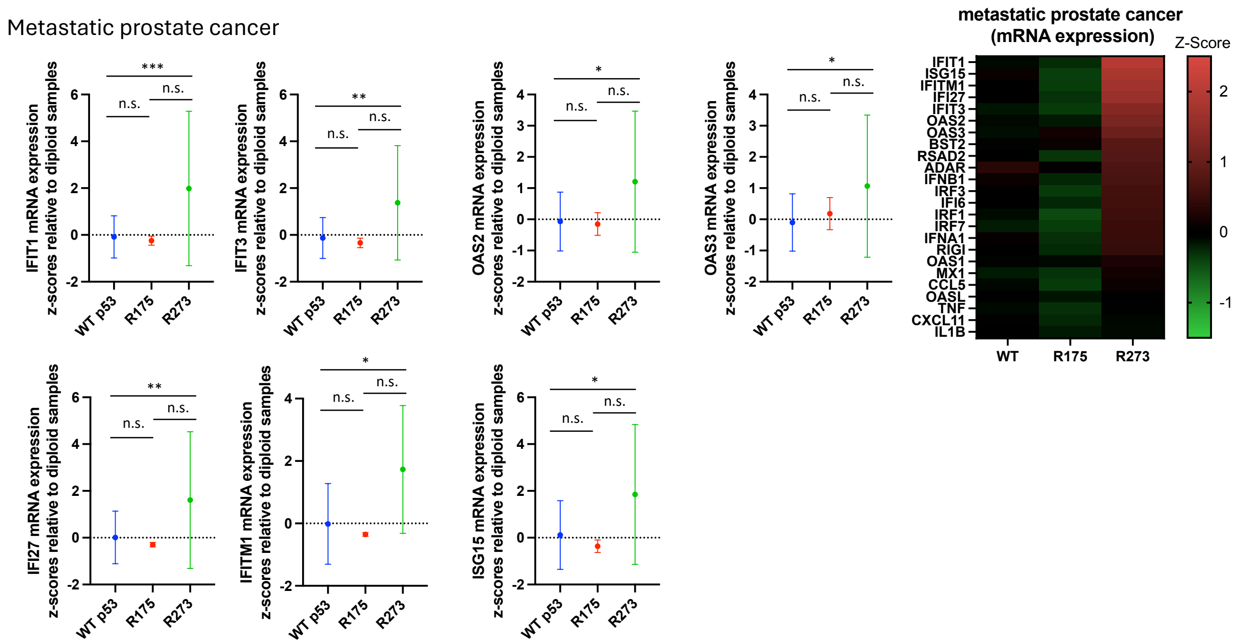

Fig. S10. Metastatic prostate cancers with mutations in the R273 residue of p53 express higher levels of cGAS-STING target genes compared to those with wild-type *TP53* or mutations in the R175 residue. Related to Fig. 10A.

mRNA expression of cGAS-STING target genes in tumors harboring either wild-type (WT) *TP53*, mutp53-R175 or mutp53-R273 in a metastatic prostate cancer dataset [5], which contains 444 samples. Shown are mean z-scores (mRNA expression relative to diploid samples) ± SD. N = 281 (WT), 5 (R175), and 9 (R273), respectively. To avoid confounding effects from different *TP53* mutations, tumors containing an additional allele of *TP53* mutation were excluded. *$P < 0.05$, **$P < 0.01$, ***$P < 0.001$, ns, not significant (two-tailed t test). Right panel: Heatmap showing mean mRNA expression levels.

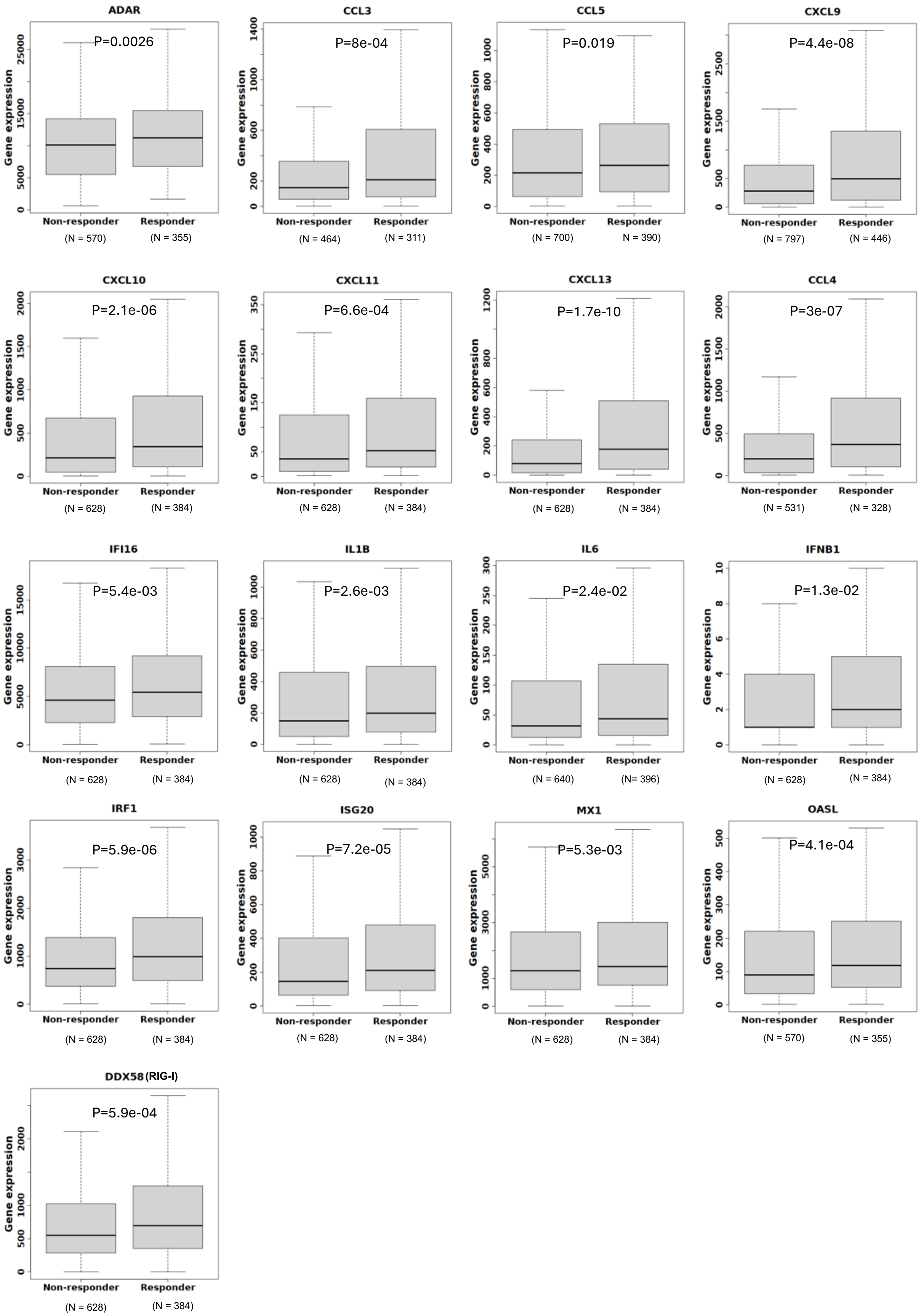
ADAR
P=0.0026
Non-responder (N = 570)
Responder (N = 355)
CCL3
P=8e-04
Non-responder (N = 464)
Responder (N = 311)
CCL5
P=0.019
Non-responder (N = 700)
Responder N = 390)
CXCL9
P=4.4e-08
Non-responder (N = 797)
Responder (N = 446)
CXCL10
P=2.1e-06
Non-responder (N = 628)
Responder (N = 384)
CXCL11
P=6.6e-04
Non-responder (N = 628)
Responder (N = 384)
CXCL13
P=1.7e-10
Non-responder (N = 628)
Responder (N = 384)
CCL4
P=3e-07
Non-responder (N = 531)
Responder (N = 328)
IFI16
P=5.4e-03
Non-responder (N = 628)
Responder (N = 384)
IL1B
P=2.6e-03
Non-responder (N = 628)
Responder (N = 384)
IL6
P=2.4e-02
Non-responder (N = 640)
Responder (N= 396)
IFNB1
P=1.3e-02
Non-responder (N = 628)
Responder (N = 384)
IRF1
P=5.9e-06
Non-responder (N = 628)
Responder (N = 384)
ISG20
P=7.2e-05
Non-responder (N = 628)
Responder (N = 384)
MX1
P=5.3e-03
Non-responder (N = 628)
Responder (N = 384)
OASL
P=4.1e-04
Non-responder (N = 570)
Responder (N = 355)
DDX58 (RIG-I)
P=5.9e-04
Non-responder (N = 628)
Responder (N = 384)
Gene expression

Fig. S11. The tumors that respond to immune checkpoint inhibitors express higher levels of cGAS-STING target genes. Related to Fig. 10F.

The boxplots of cGAS-STING target gene expression shown in Fig. 10F. Gene expression in pretreated tumors was stratified according to their response to immune checkpoint inhibitors. Analysis was performed in ROC Plotter including all types of cancer and any immune checkpoint inhibitors. The bottom labels show the sample numbers (N) for each group.

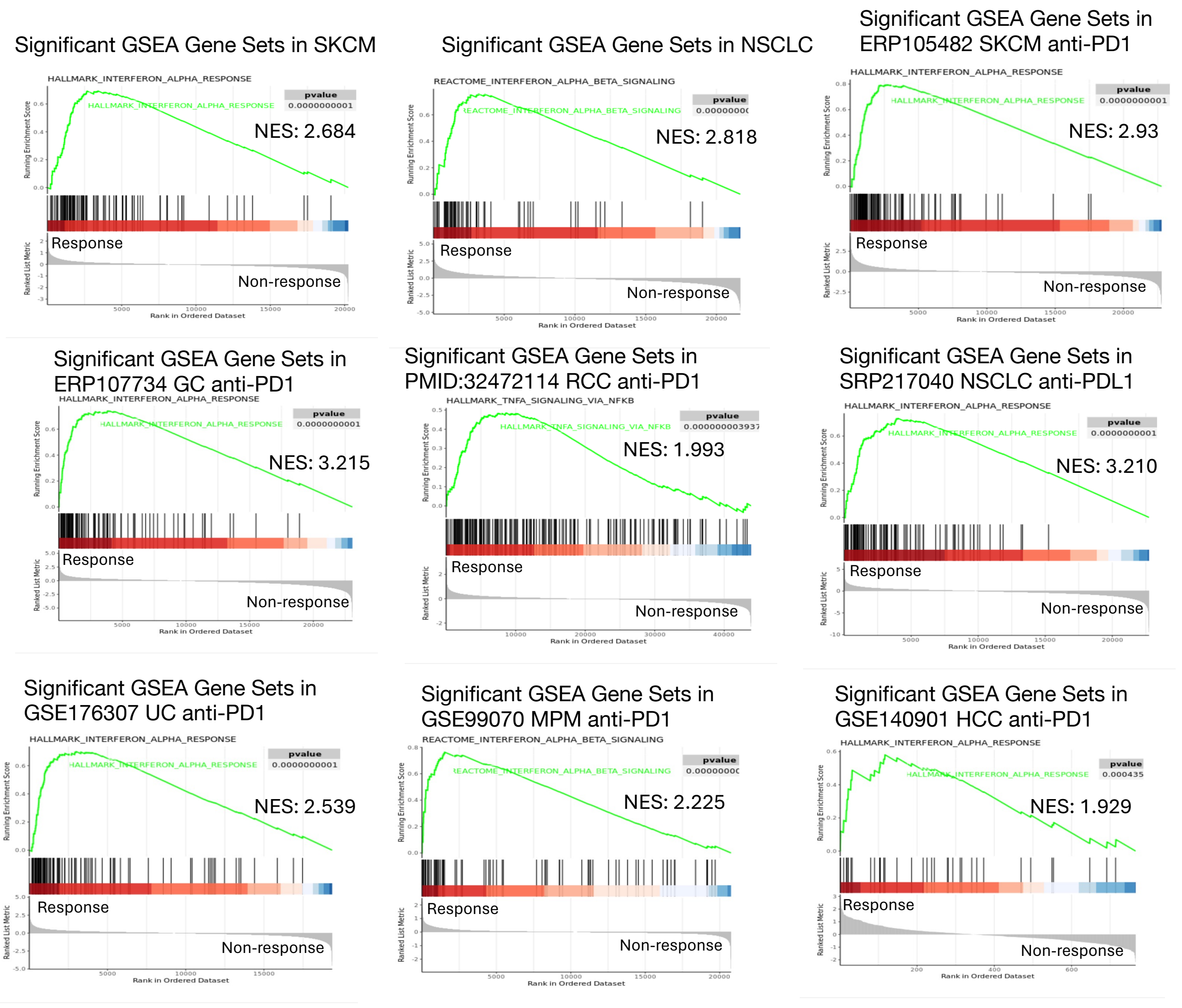

Fig. S12. Significant enrichment of Interferon-α response genes in responders across cancer types and immune checkpoint inhibitors. Related to Fig. 10G and 10H.

GSEA analyses of DEGs (differentially expressed genes) in the responders vs. non-responders to immune checkpoint inhibitors. Hallmark_Interferon_alpha_response gene set is highly enriched in various datasets across different types of cancer. NES: normalized enrichment score; SKCM: skin cutaneous melanoma; NSCLC: non-small cell lung cancer; GC: gastric cancer; RCC: renal cell carcinoma; UC: urothelial carcinoma; MPM: malignant pleural mesothelioma; HCC: hepatocellular carcinoma.

Supplementary Table S1

Key resources table

REAGENT or RESOURCE	SOURCE	IDENTIFIER
Antibodies		
Anti-E2F1	Cell Signaling	Cat# 3742, RRID:AB_2096936
Anti-TopBP1 mouse monoclonal	BD Transduction Laboratories	Cat# 611875, RRID:AB_399355
Anti-TopBP1 (BL893) rabbit polyclonal	Bethyl Laboratories	Cat# A300-111A, RRID:AB_2272050
Anti-phospho-TopBP1 (S1159)	Abgent Antibody	Cat# AP3774a, RRID:AB_11134625
Anti-Treslin	Bethyl Laboratories	Cat# A303-472A, RRID:AB_10953949
Anti-p-Akt (S473)	Cell Signaling	Cat# 4060, RRID:AB_2315049
Anti-Akt (pan)	Cell Signaling	Cat# 4691, RRID:AB_915783
Anti-GAPDH (6C5)	Santa Cruz Biotechnology	Cat# sc-32233, RRID:AB_627679
Anti-Histone γ-H2AX (20E3)	Cell Signaling	Cat# 9718, RRID:AB_2118009
Anti-BrdU (purified mouse anti-BrdU) (Clone B44)	BD Transduction Laboratories	Cat# 347580, RRID:AB_400326
Anti-BrdU (purified rat monoclonal anti-BrdU)	Abcam	Cat# ab6326, RRID:AB_305426
Anti-p53 (7F5)	Santa Cruz Biotechnology	Cat# sc-2527, RRID:AB_628082
Anti-pS10-Histone H3	Cell Signaling	Cat# 9701, RRID:AB_331535
Anti-Histone H3	Cell Signaling	Cat# 9715, RRID:AB_331558
Anti-PCNA (PC10)	Santa Cruz Biotechnology	Cat# sc-56, RRID:AB_628110
Anti-MRE11 (18)	Santa Cruz Biotechnology	Cat# sc-135992, RRID:AB_2145244
Anti-MCM2	Abnova	Cat# H00004171-A01, RRID:AB_463194
Anti-cGAS (D1D3G)	Cell Signaling	Cat# 15102, RRID:AB_2732795
Anti-p-STING (S336)	Cell Signaling	Cat# 19781, RRID:AB_2737062
Anti-STING (D2P2F)	Cell Signaling	Cat# 19781, RRID:AB_2732796
Anti-p-TBK1/NAK (S172)	Cell Signaling	Cat# 5483, RRID:AB_10693472
Anti-TBK1/NAK (E8I3G)	Cell Signaling	Cat# 38066, RRID:AB_2827657
Anti-ISG15 (2202)	Cell Signaling	Cat# 2758, RRID:AB_2126200
Anti-p-eIF2α (S51)	Cell Signaling	Cat# 9721, RRID:AB_330951

Anti-eIF2α (G12)	Cell Signaling	Cat# 9722, RRID:AB_2230924
Myc-Trap agarose beads	Chromoteck	Cat# yta, RRID: AB_2631369
Goat anti-mouse IgG (H+L) Texas-Red-X conjugated	Invitrogen	Cat# T6390, RRID:AB_2556778
Chicken anti-rat IgG (H+L) Alexa Fluor 488 conjugated	Life Technologies	Cat# A21470, RRID:AB_10561519
Rat anti-mouse CD279 (PD-1) antibody (clone 29F.1A12) – Purified *in vivo* GOLD™ Functional Grade	Leinco Technologies	Cat# P377, RRID:AB_2737558
Rat IgG2a isotype control – Purified *in vivo* GOLD™ Functional Grade	Leinco Technologies	Cat# I-1177, RRID:AB_2737530
Rabbit monoclonal anti-CD8	Cell Signaling	Cat# 98941, RRID:AB_2756376
Goat polyclonal anti-mouse Granzyme B	R&D Systems	Cat# AF1865, RRID:AB_2294988
Experimental models: Cell lines		
H1299	ATCC	Cat# CRL-5803, RRID:CVCL_0060
MDA-MB468	ATCC	Cat# HTB-132, RRID:CVCL_0419
SKBR3	ATCC	Cat# HTB-30, RRID:CVCL_0033
TOV-112D	ATCC	Cat# CRL-3593, RRID:CVCL_3612
MDAH-2774	ATCC	Cat# CRL-10303, RRID:CVCL_0420
C33A	ATCC	Cat# HTB-31, RRID:CVCL_1094
MDA-MB-231	ATCC	Cat# HTB-26, RRID:CVCL_0062
4T1	ATCC	Cat# CRL-2539, RRID:CVCL_0125
Experimental models: Organisms/strains		
BALB/cJ	Jackson Laboratory	Strain Number: 000651
Oligonucleotides		
cGAS forward primer: AGGAAGCAACTACGACTAAAGCC	This paper (IDT)	N/A
cGAS reverse primer: CGATGTGAGAGAAGGATAGCCG	This paper (IDT)	N/A
STING forward primer: CCTGAGTCTCAGAACAACTGCC	This paper (IDT)	N/A
STING reverse primer: GGTCTTCAAGCTGCCCACAGTA	This paper (IDT)	N/A
IFNA1 forward primer: AGAAGGCTCCAGCCATCTCTGT	This paper (IDT)	N/A
IFNA1 reverse primer: TGCTGGTAGAGTTCGGTGCAGA	This paper (IDT)	N/A
IFNB1 forward primer: CTTGGATTCCTACAAAGAAGCAGC	This paper (IDT)	N/A
IFNB1 reverse primer: TCCTCCTTCTGGAACTGCTGCA	This paper (IDT)	N/A

IFN-γ forward primer: GAGTGTGGAGACCATCAAGGAAG	This paper (IDT)	N/A
IFN-γ reverse primer: TGCTTTGCGTTGGACATTCAAGTC	This paper (IDT)	N/A
CCL5 forward primer: CCTGCTGCTTTGCCTACATTGC	This paper (IDT)	N/A
CCL5 reverse primer: ACACACTTGGCGGTTCTTTCGG	This paper (IDT)	N/A
ISG15 forward primer: CTCTGAGCATCCTGGTGAGGAA	This paper (IDT)	N/A
ISG15 reverse primer: AAGGTCAGCCAGAACAGGTCGT	This paper (IDT)	N/A
GAPDH forward primer: ATTGGGCGCCTGGTCACCAGGGCTG	This paper (IDT)	N/A
GAPDH reverse primer: AAATGAGCCCCAGCCTTCTCCATG	This paper (IDT)	N/A
Recombinant DNA		
pLKO.1-shScramble	Sarbassov et al. [6]	Addgene Plasmid #136035
pLKO.1-shp53	Godar et al. [7]	Addgene Plasmid #19119
pCMV-Neo-Bam	Baker et al. [8]	Addgene Plasmid #16440
pCMV-Neo-Bam p53 wt	Baker et al. [8]	Addgene Plasmid #16434
pCMV-Neo-Bam mutp53-R175H	Baker et al. [8]	Addgene Plasmid #16436
pCMV- Neo-Bam mutp53-R273H	Baker et al. [8]	Addgene Plasmid #16439
pCMV-Neo-Bam mutp53-R249S	Baker et al. [8]	Addgene Plasmid #16438
pCMV- Neo-Bam mutp53-R248W	Baker et al. [8]	Addgene Plasmid #16437
pcDNA5/TO-Treslin-Myc	Kumagai et al. [9]	N/A

Supplementary References:

1. Bartha A, Gyorffy B. TNMplot.com: A Web Tool for the Comparison of Gene Expression in Normal, Tumor and Metastatic Tissues. *Int J Mol Sci* **22**, (2021).

2. Chandrashekar DS*, et al.* UALCAN: An update to the integrated cancer data analysis platform. *Neoplasia* **25**, 18-27 (2022).

3. Curtis C*, et al.* The genomic and transcriptomic architecture of 2,000 breast tumours reveals novel subgroups. *Nature* **486**, 346-352 (2012).

4. Bottomly D*, et al.* Integrative analysis of drug response and clinical outcome in acute myeloid leukemia. *Cancer Cell* **40**, 850-864 e859 (2022).

5. Abida W*, et al.* Genomic correlates of clinical outcome in advanced prostate cancer. *Proc Natl Acad Sci U S A* **116**, 11428-11436 (2019).

6. Sarbassov DD, Guertin DA, Ali SM, Sabatini DM. Phosphorylation and regulation of Akt/PKB by the rictor-mTOR complex. *Science* **307**, 1098-1101 (2005).

7. Godar S*, et al.* Growth-inhibitory and tumor- suppressive functions of p53 depend on its repression of CD44 expression. *Cell* **134**, 62-73 (2008).

8. Baker SJ, Markowitz S, Fearon ER, Willson JK, Vogelstein B. Suppression of human colorectal carcinoma cell growth by wild-type p53. *Science* **249**, 912-915 (1990).

9. Kumagai A, Shevchenko A, Shevchenko A, Dunphy WG. Direct regulation of Treslin by cyclin-dependent kinase is essential for the onset of DNA replication. *J Cell Biol* **193**, 995-1007 (2011).
