# Supplementary material for: Mutant p53 variants differentially impact replication initiation and activate cGAS-STING to affect immune checkpoint inhibition
Source: Commun Biol. 2025 Nov 5;8:1522. doi: 10.1038/s42003-025-09050-3 (PMC12589595; doi:10.1038/s42003-025-09050-3)
Supplement: Supplementary file 3 — Description of Additional Supplementary Files [file 42003_2025_9050_MOESM3_ESM.pdf]

Description of Additional Supplementary Files

File name: Supplementary Data 1
Description: Uncropped blot images.

File name: Supplementary Data 2
Description: The numeric source data for the figures and supplementary figures in the paper.
