# Supplementary material for: Mutant p53 variants differentially impact replication initiation and activate cGAS-STING to affect immune checkpoint inhibition
Source: Commun Biol. 2025 Nov 5;8:1522. doi: 10.1038/s42003-025-09050-3 (PMC12589595; doi:10.1038/s42003-025-09050-3)

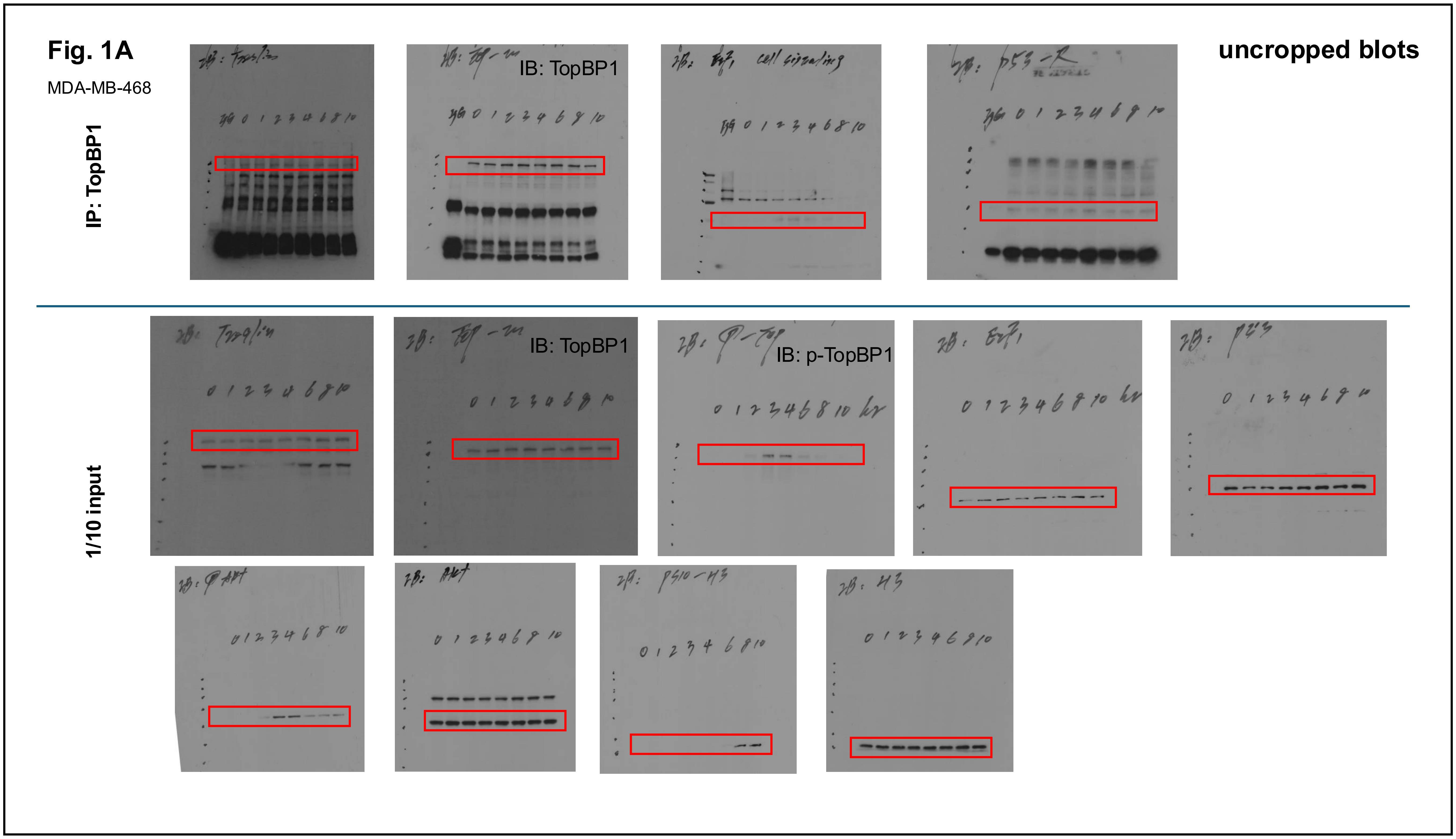
Fig. 1A
MDA-MB-468
uncropped blots
IP: TopBP1
IB: TopBP1
1/10 input
IB: TopBP1
IB: p-TopBP1

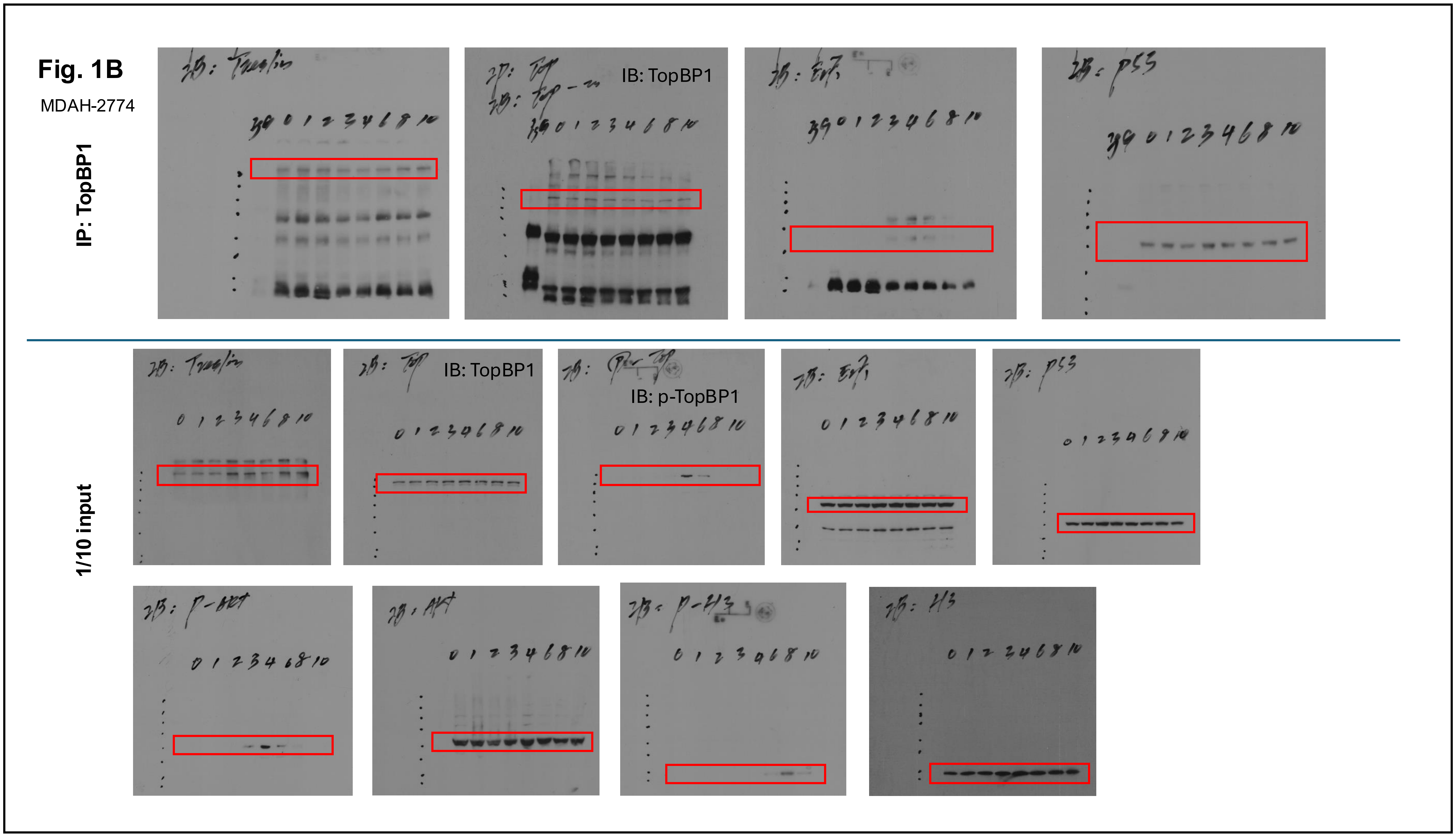
Fig. 1B
MDAH-2774
IP: TopBP1
IB: TopBP1
1/10 input
IB: TopBP1
IB: p-TopBP1

Fig. 1C

SKBR3

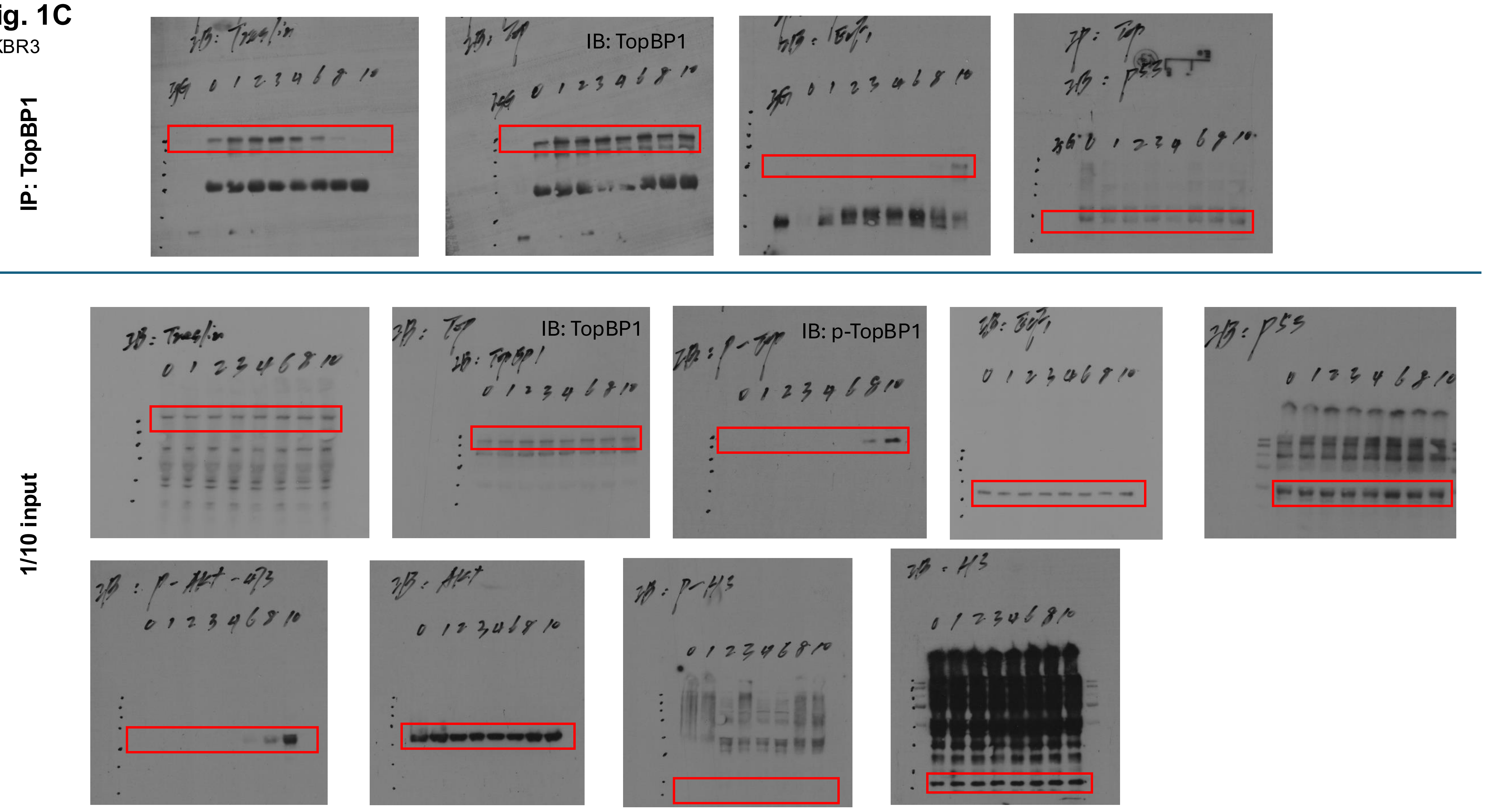

Fig. 1D

TOV-112D

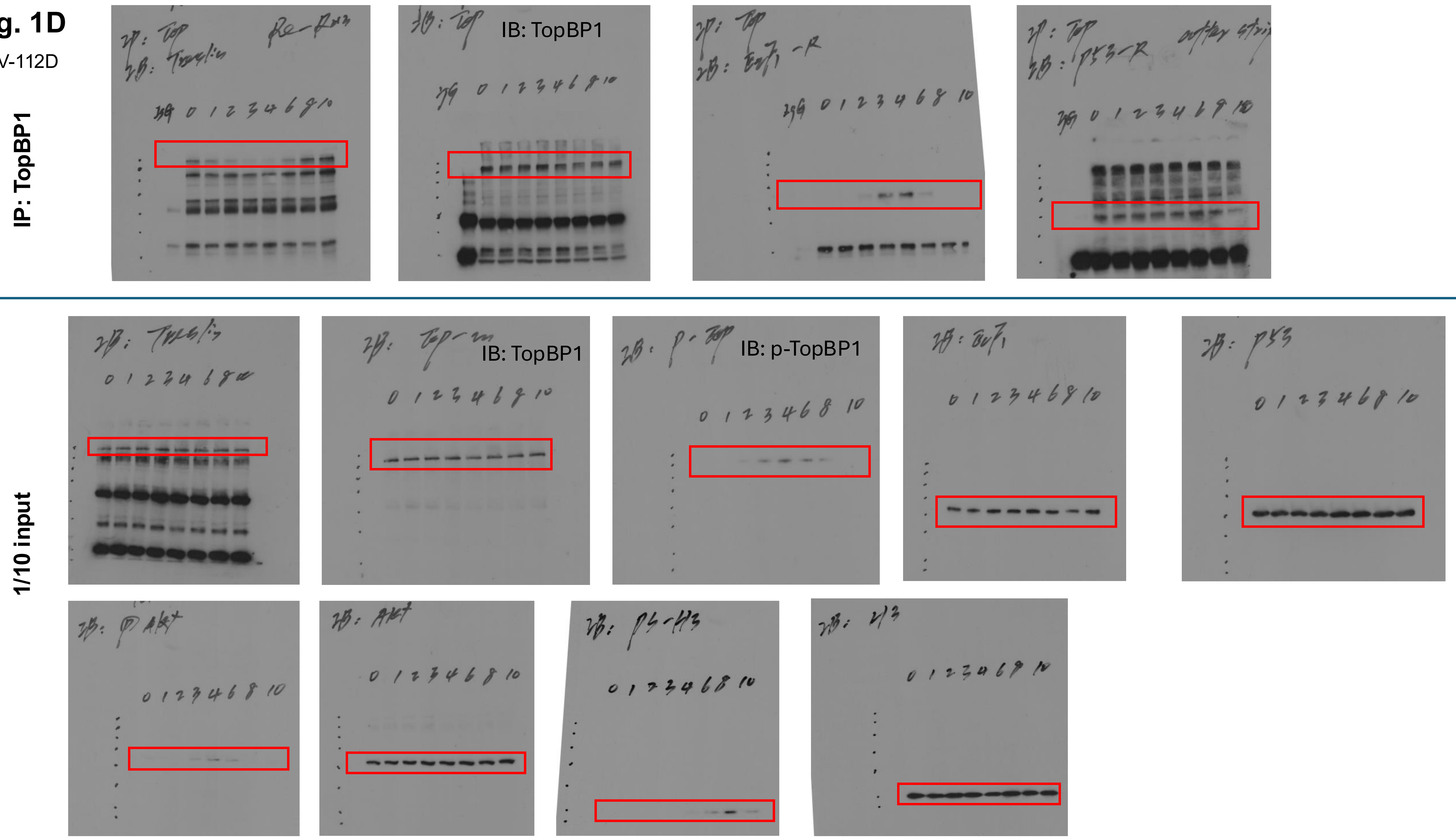

Fig. 2A

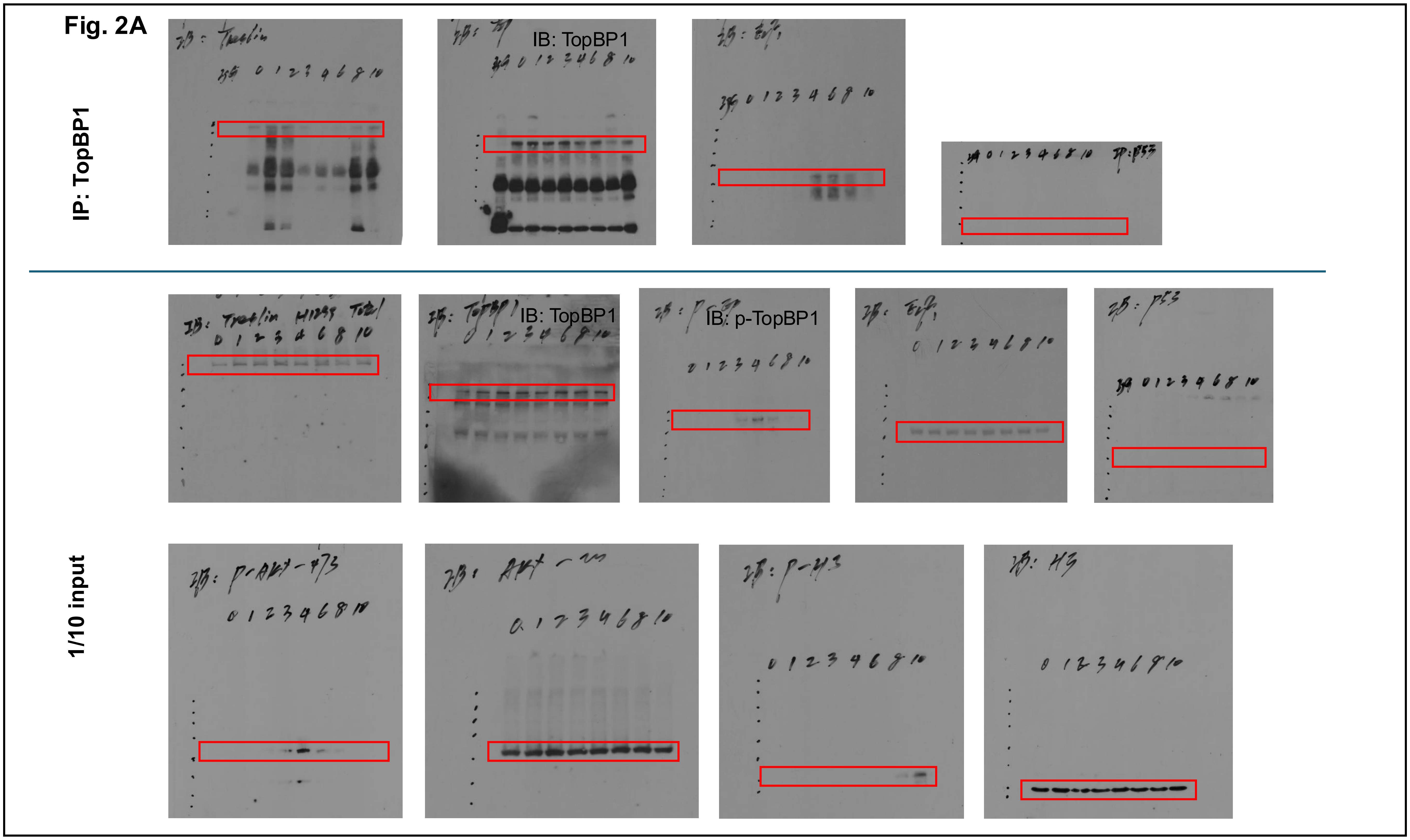
IP: TopBP1
IB: TopBP1
IB: TopBP1
IB: p-TopBP1
1/10 input

Fig. 2B

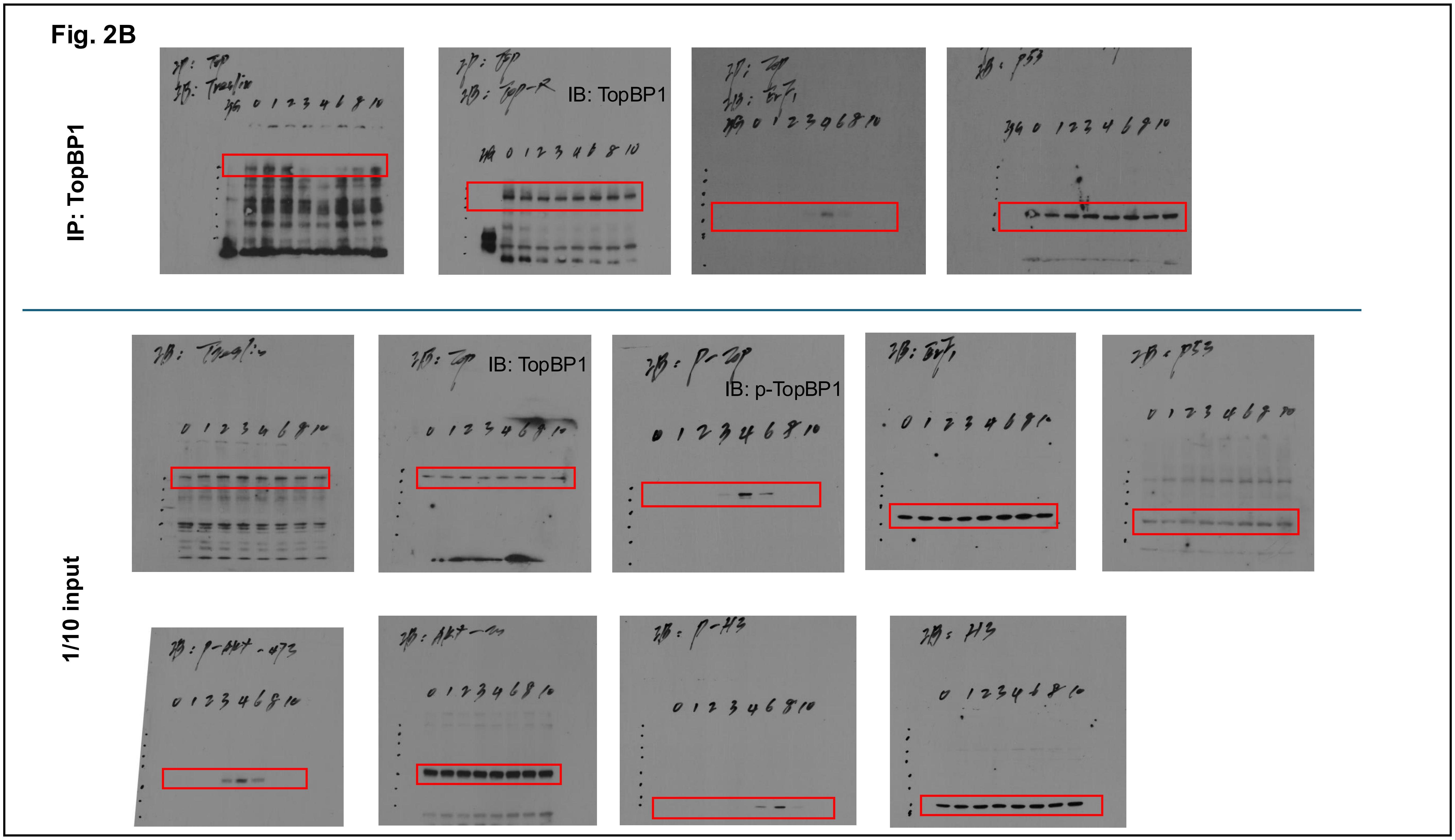
IP: TopBP1
IB: TopBP1
IB: TopBP1
IB: p-TopBP1
1/10 input

Fig. 2C

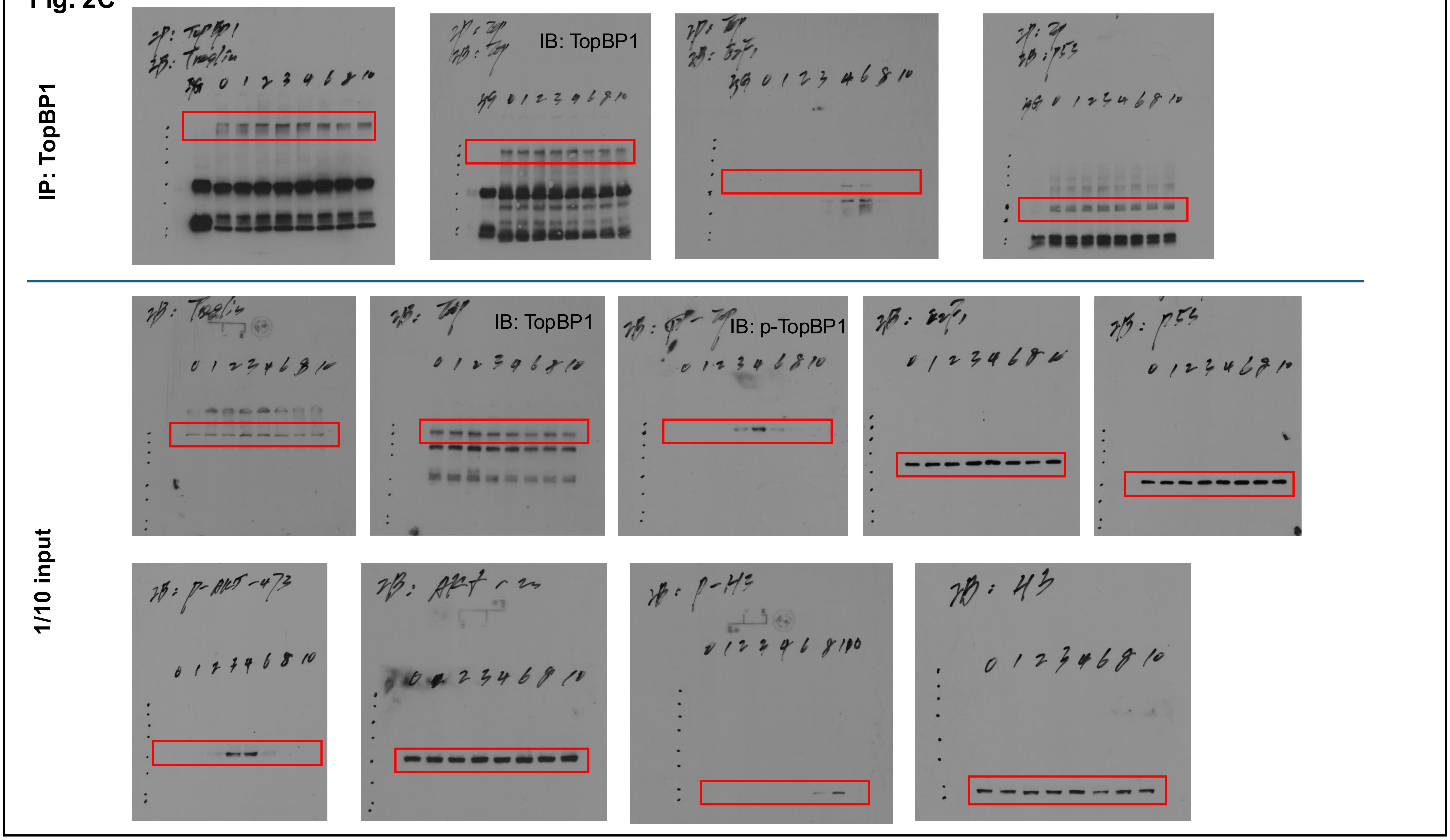
IP: TopBP1
IB: TopBP1
1/10 input
IB: TopBP1
IB: p-TopBP1

Fig. 2D

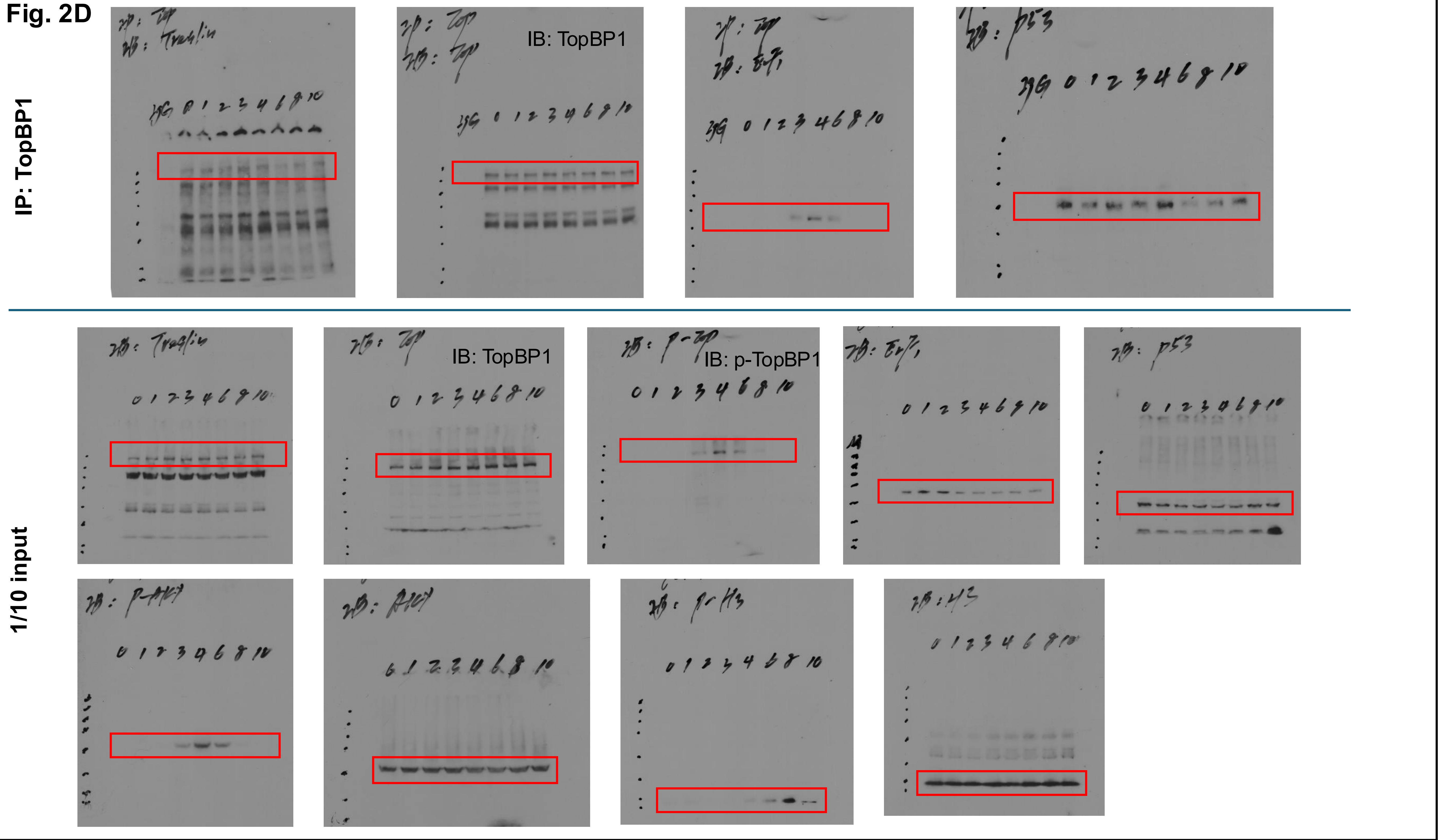
IP: TopBP1
IB: TopBP1
1/10 input
IB: TopBP1
IB: p-TopBP1

Fig. 2E

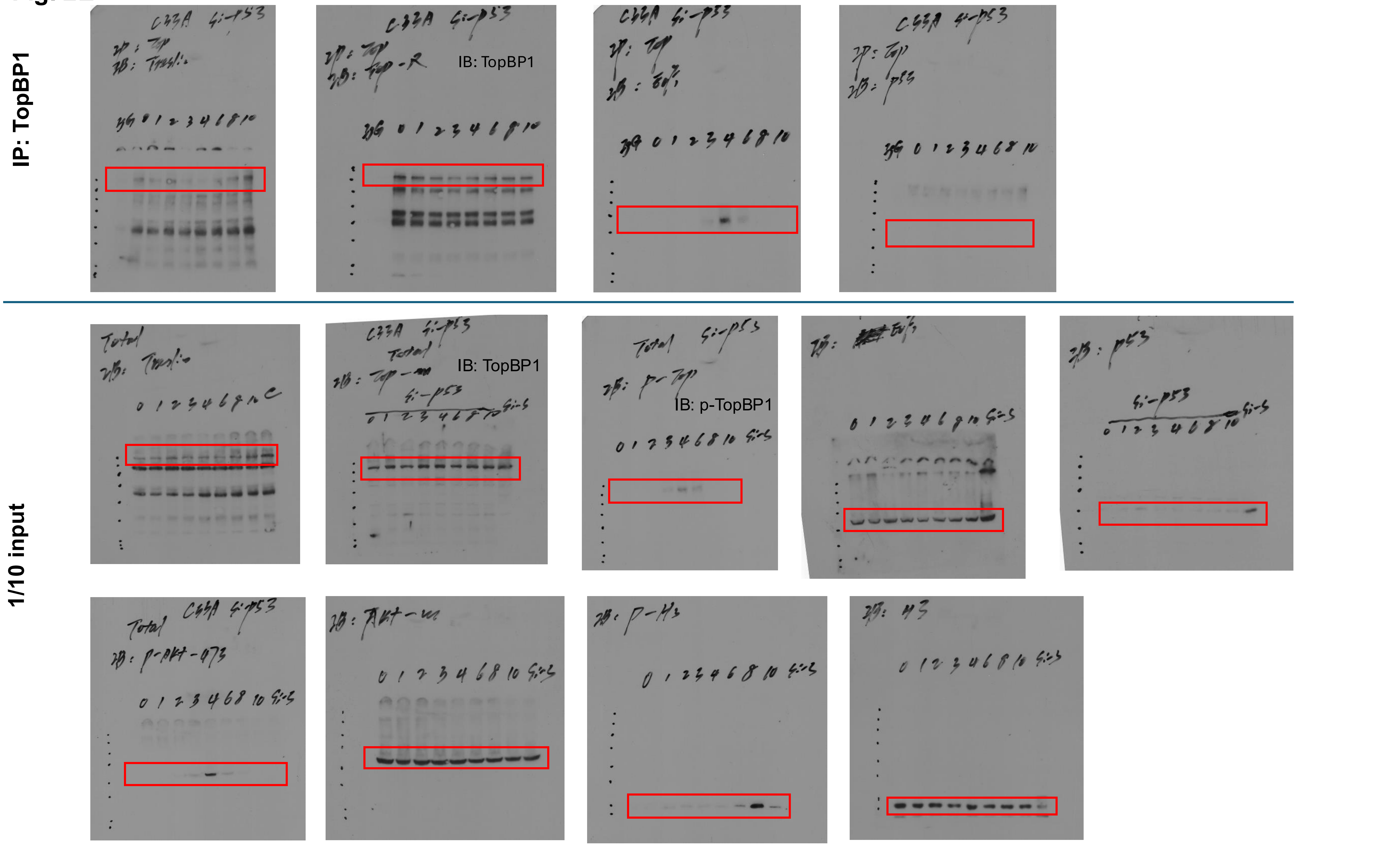
IP: TopBP1
IB: TopBP1
1/10 input
IB: TopBP1
IB: p-TopBP1

Fig. 2F

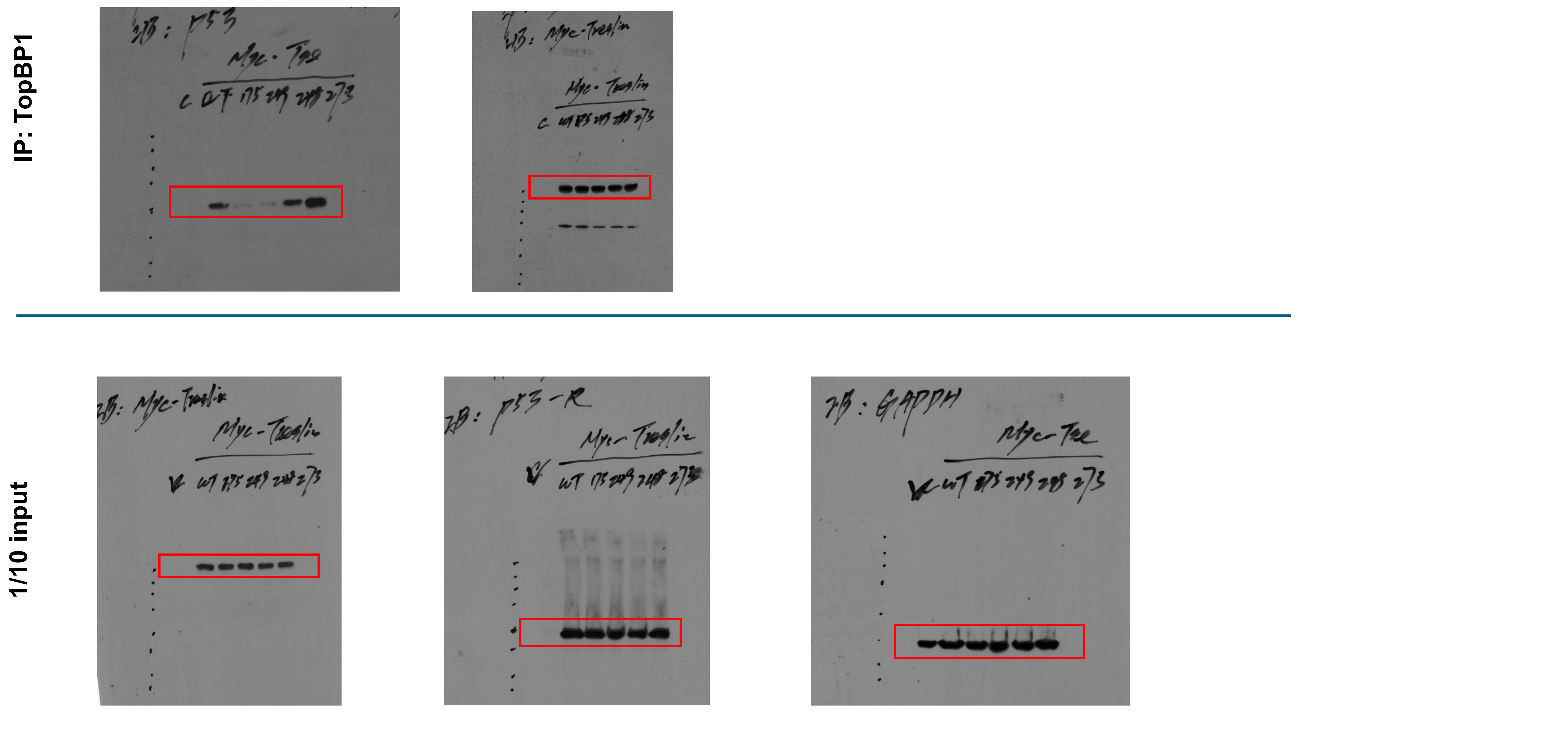
IP: TopBP1
1/10 input

Fig. 3B

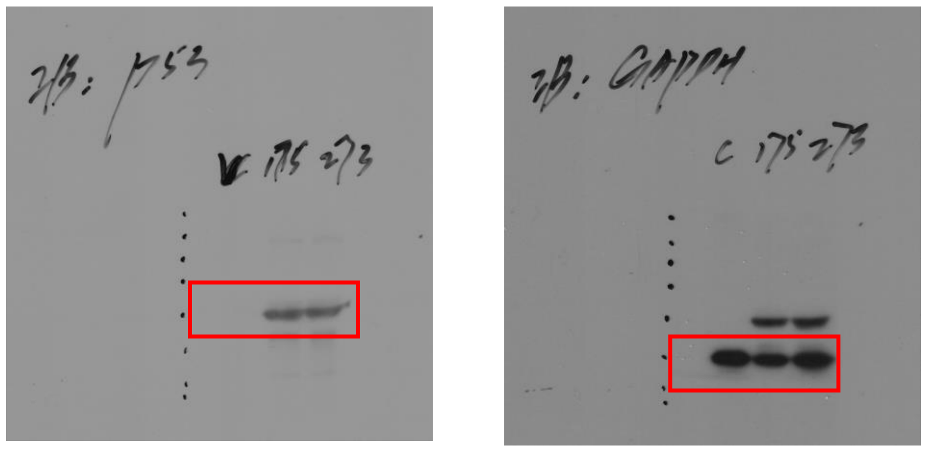

Fig. 3E

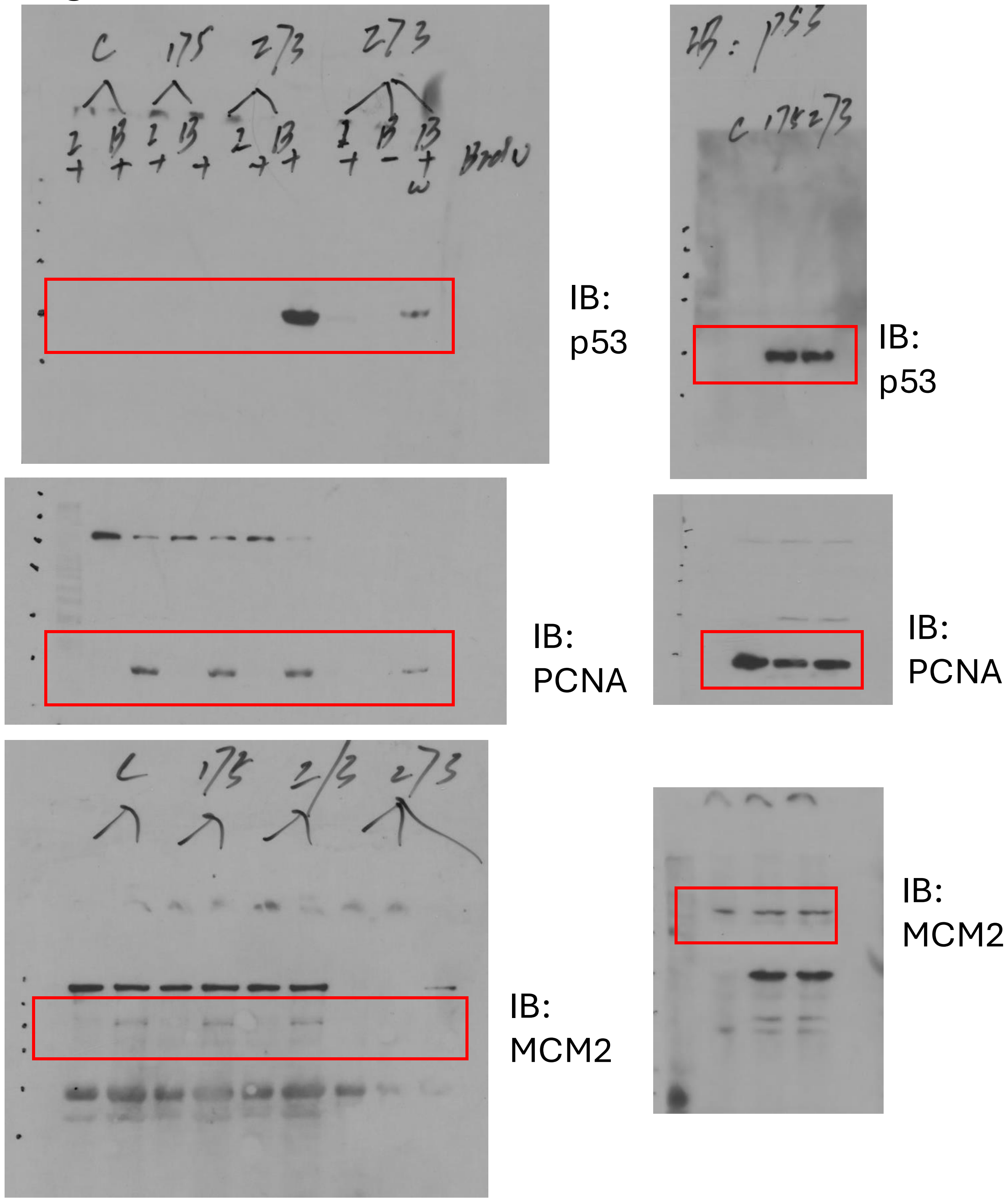

Fig. 4A

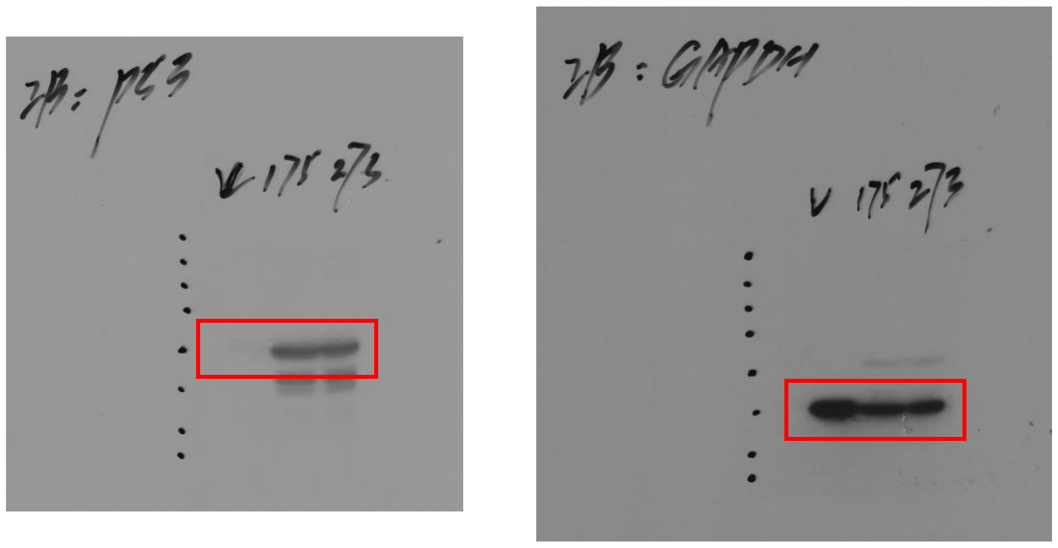

Fig. 5A

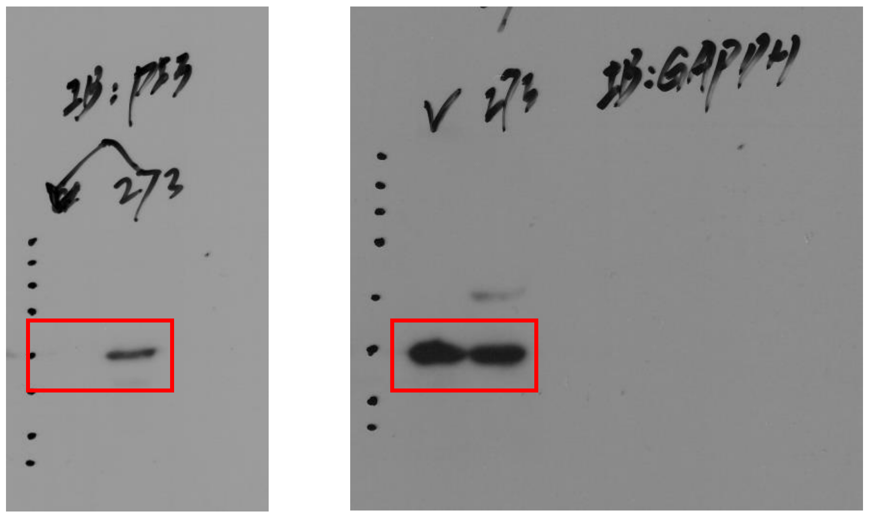

Fig. 6

Fig. 6C

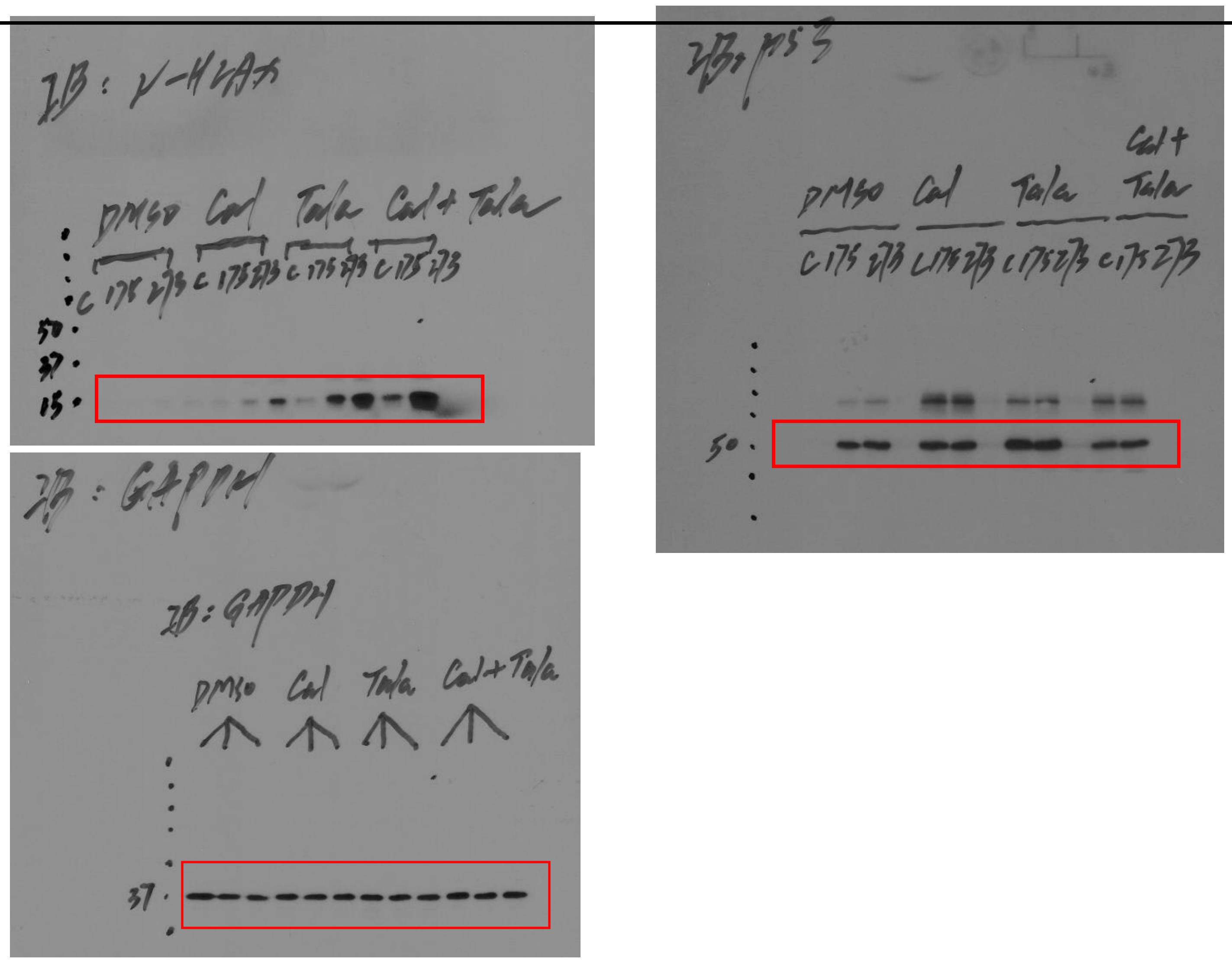

Fig. 6A

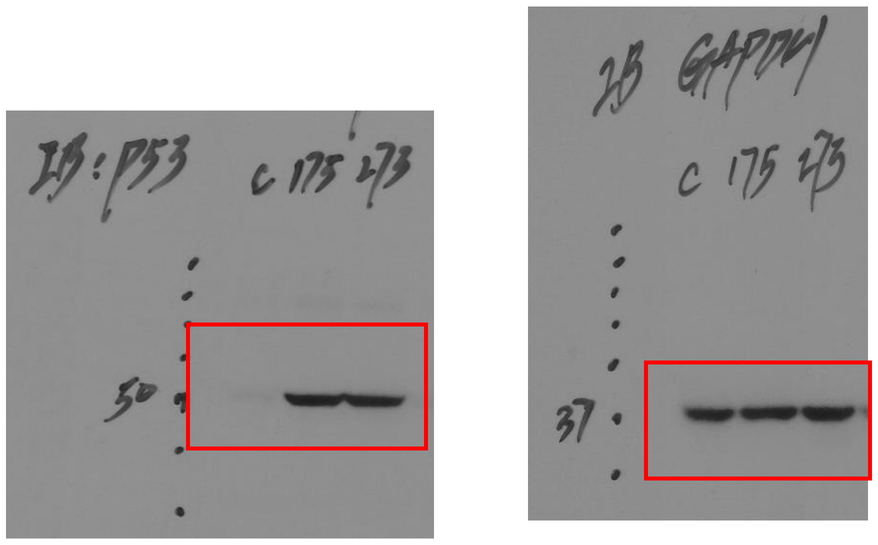

Fig. 6B

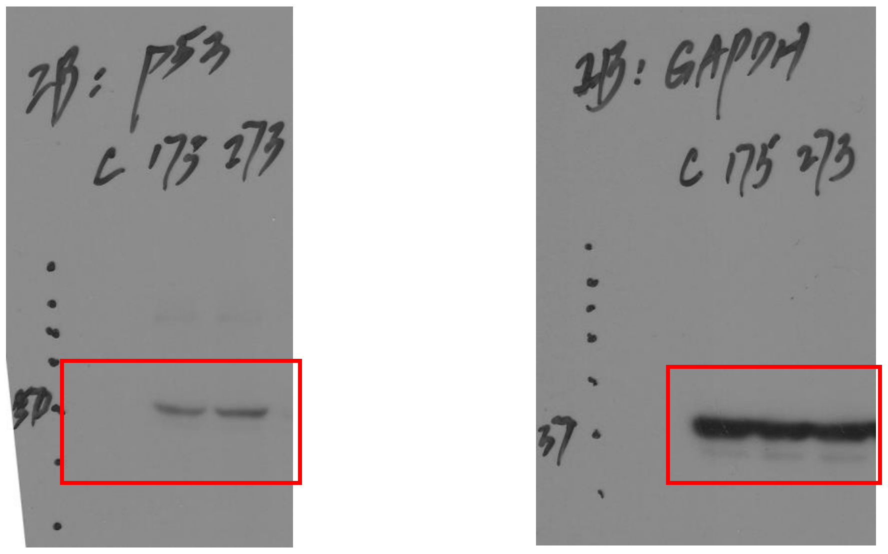

Fig. 6D

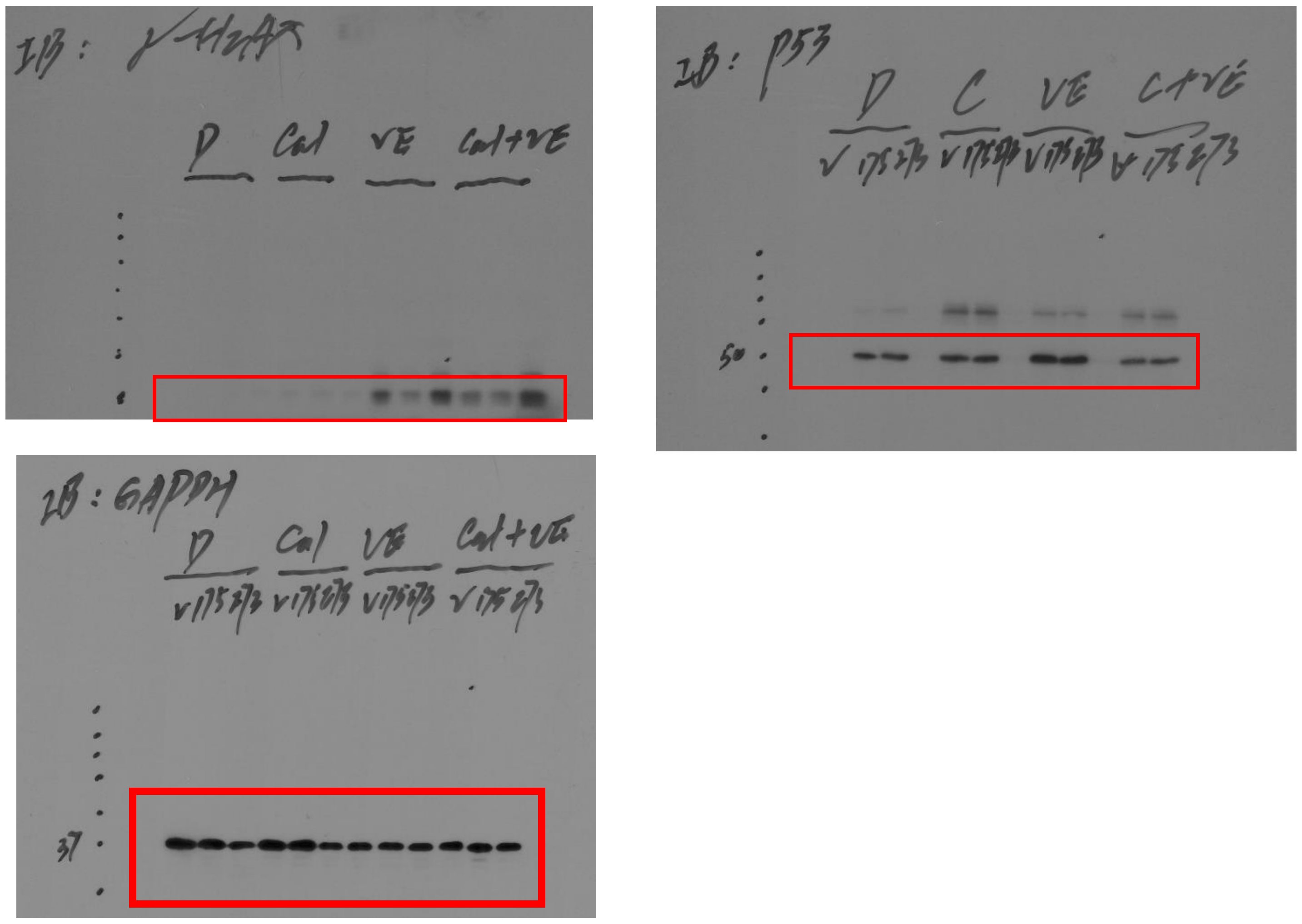

Fig. 7B

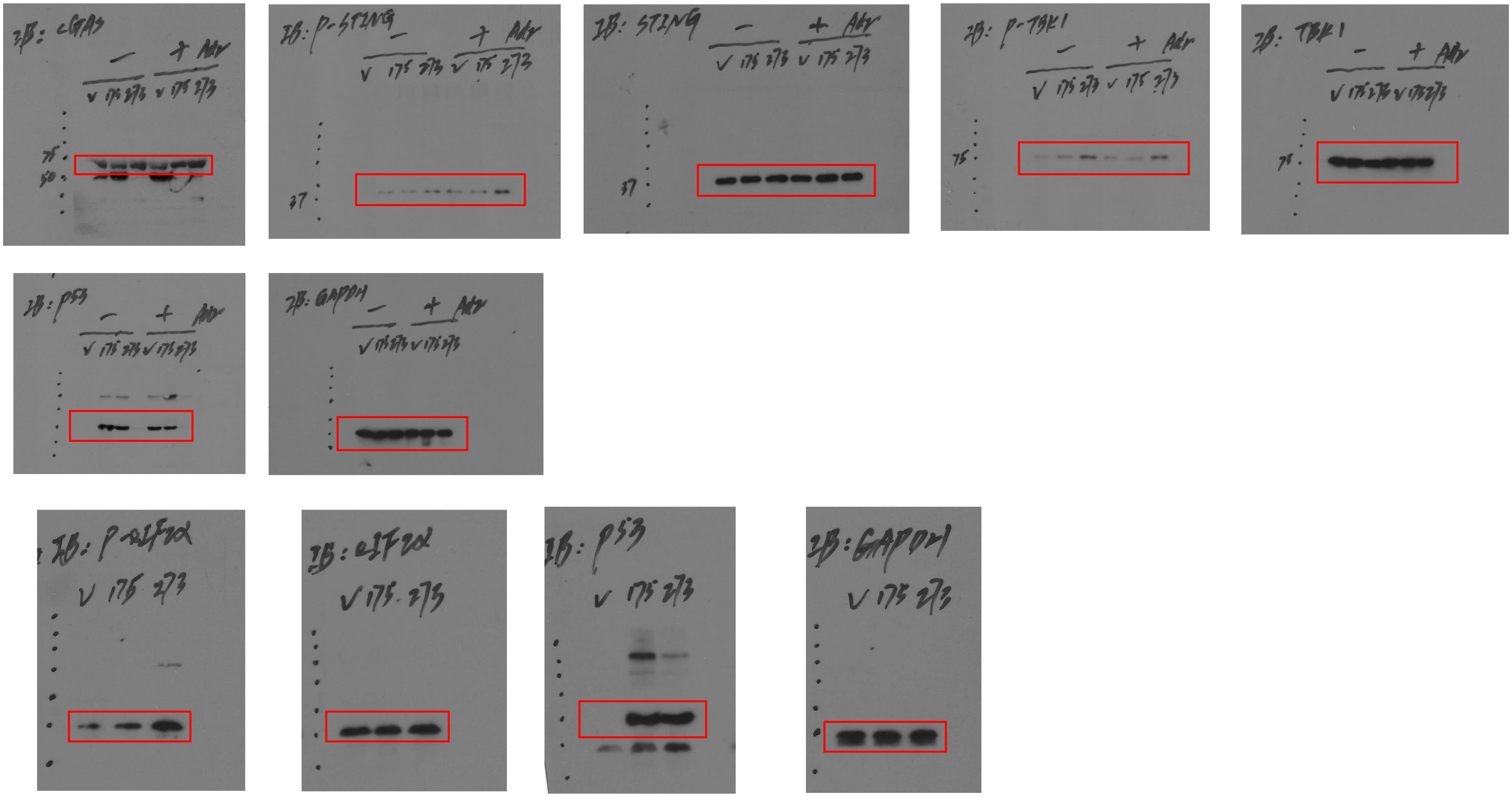
IB: cGAS
IB: p-STING
IB: STING
IB: p-TBK1
IB: TBK1
IB: p53
IB: GAPDH
IB: p53
IB: GAPDH

Fig. 7D

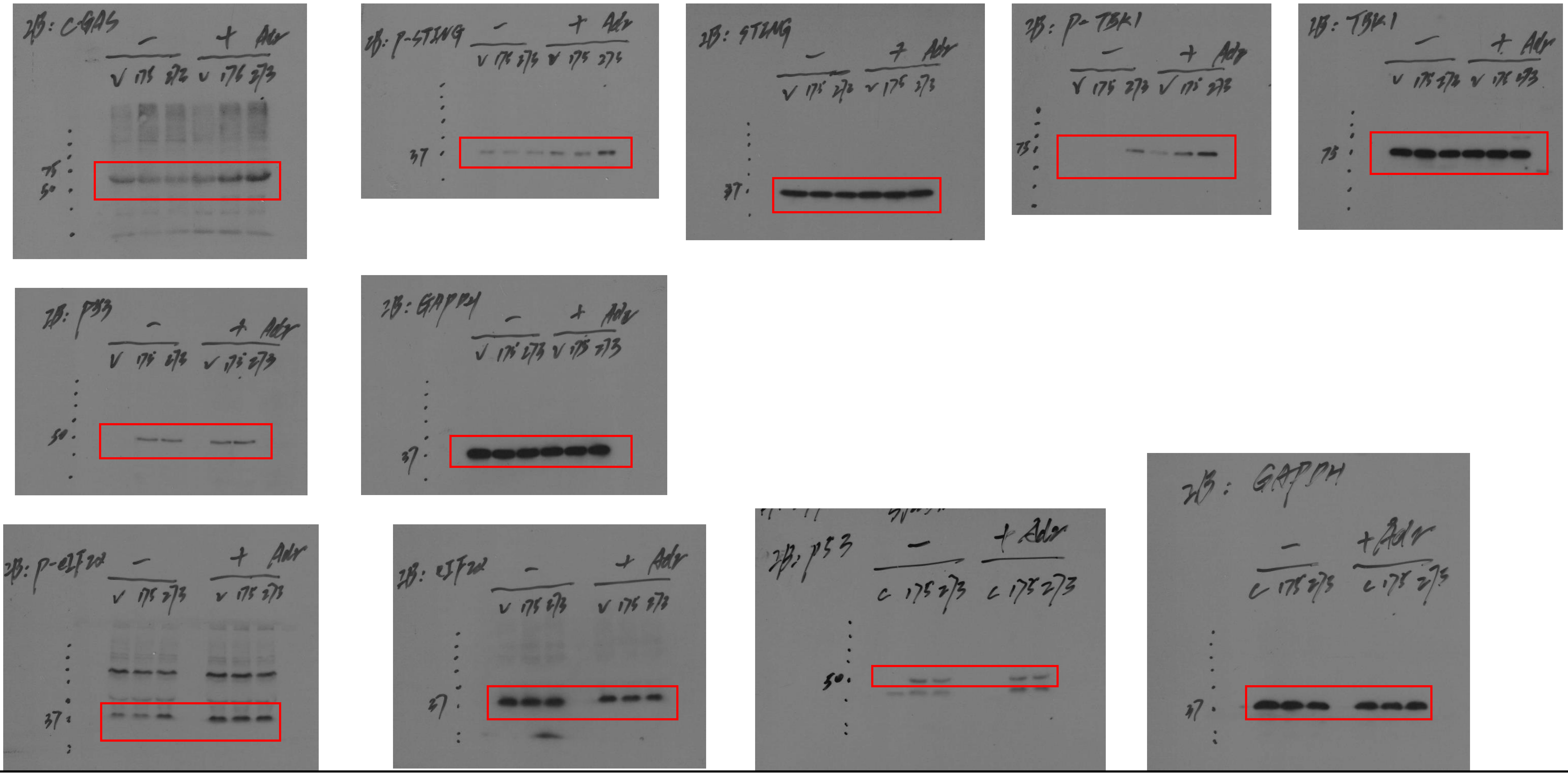
IB: cGAS
IB: p-STING
IB: STING
IB: p-TBK1
IB: TBK1
IB: p53
IB: GAPDH
IB: p53
IB: GAPDH

Fig. 7E

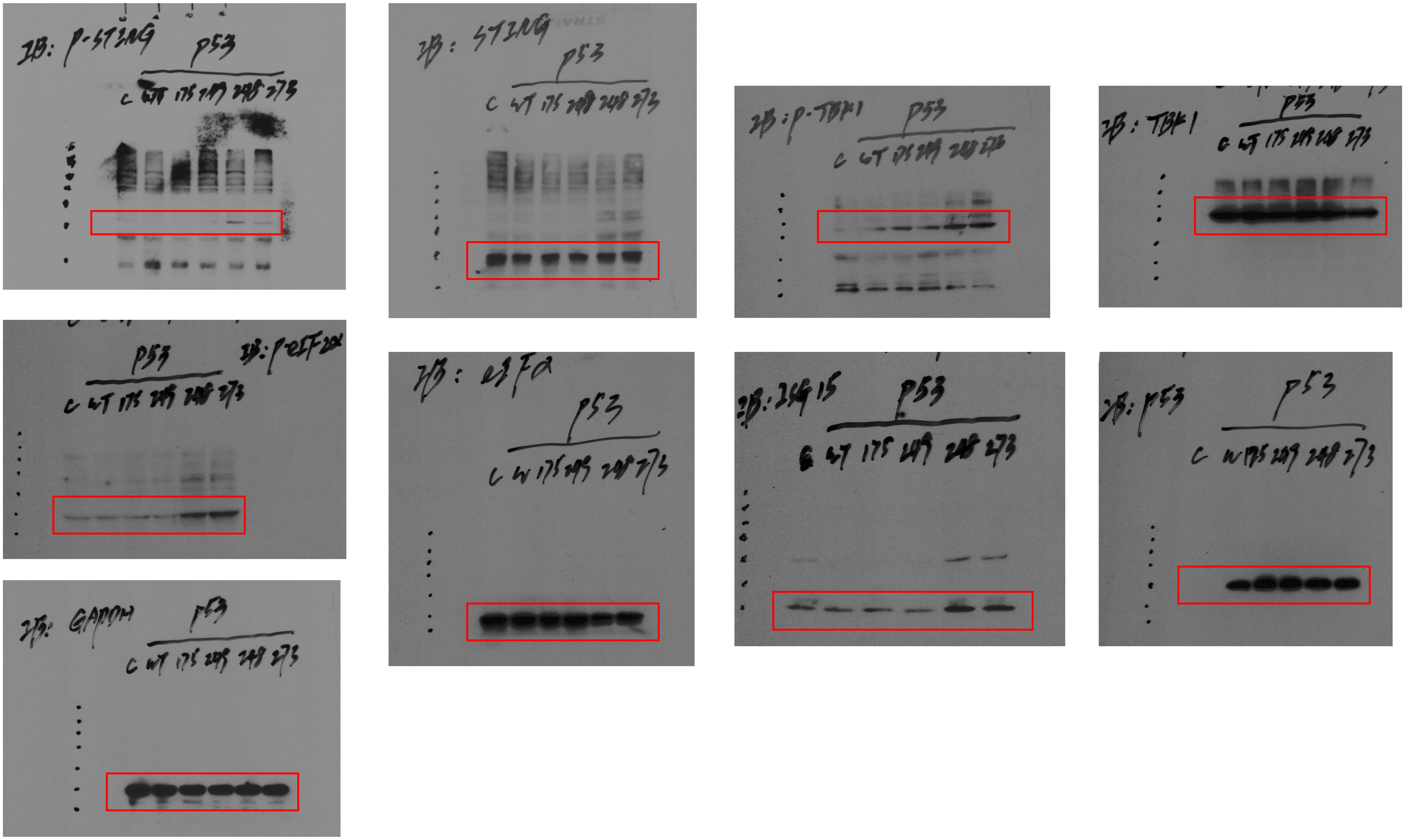

Fig. 7F

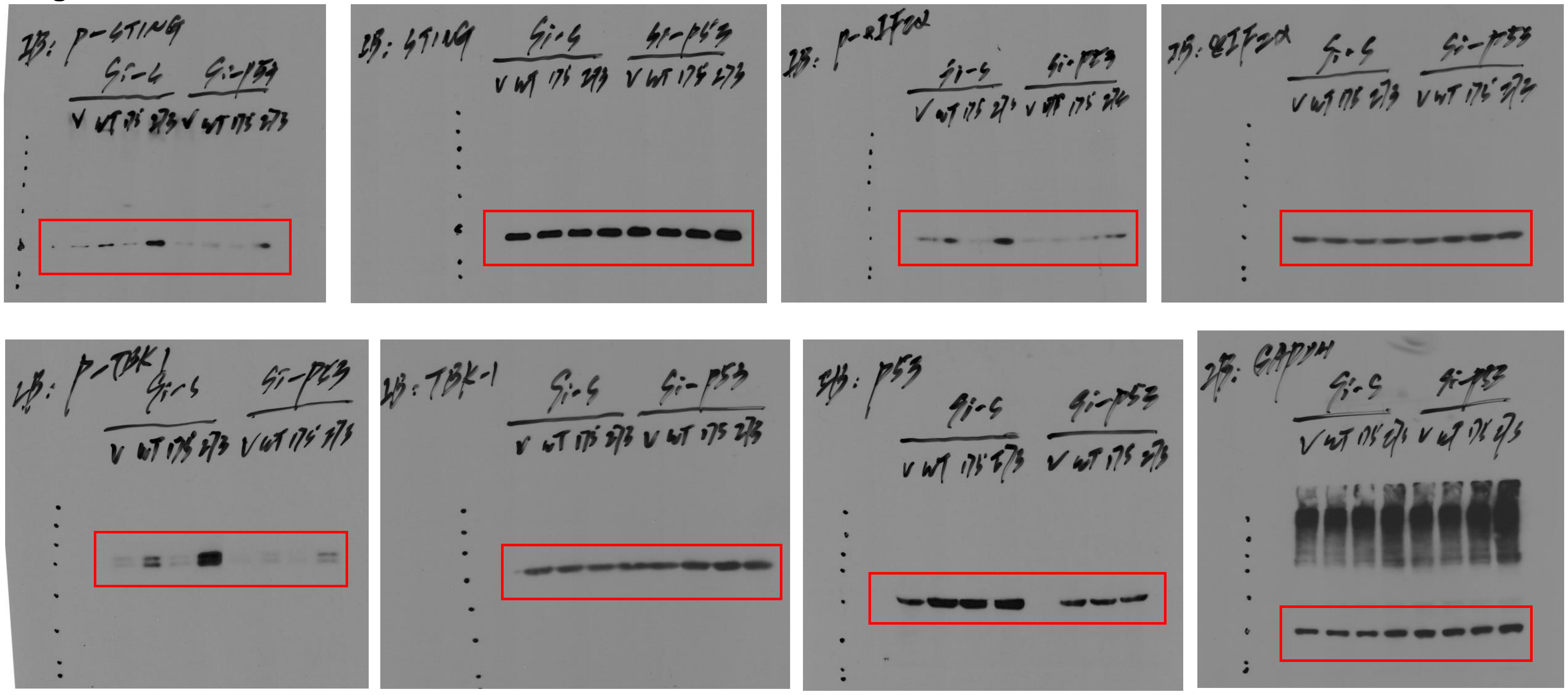

Fig. 8A

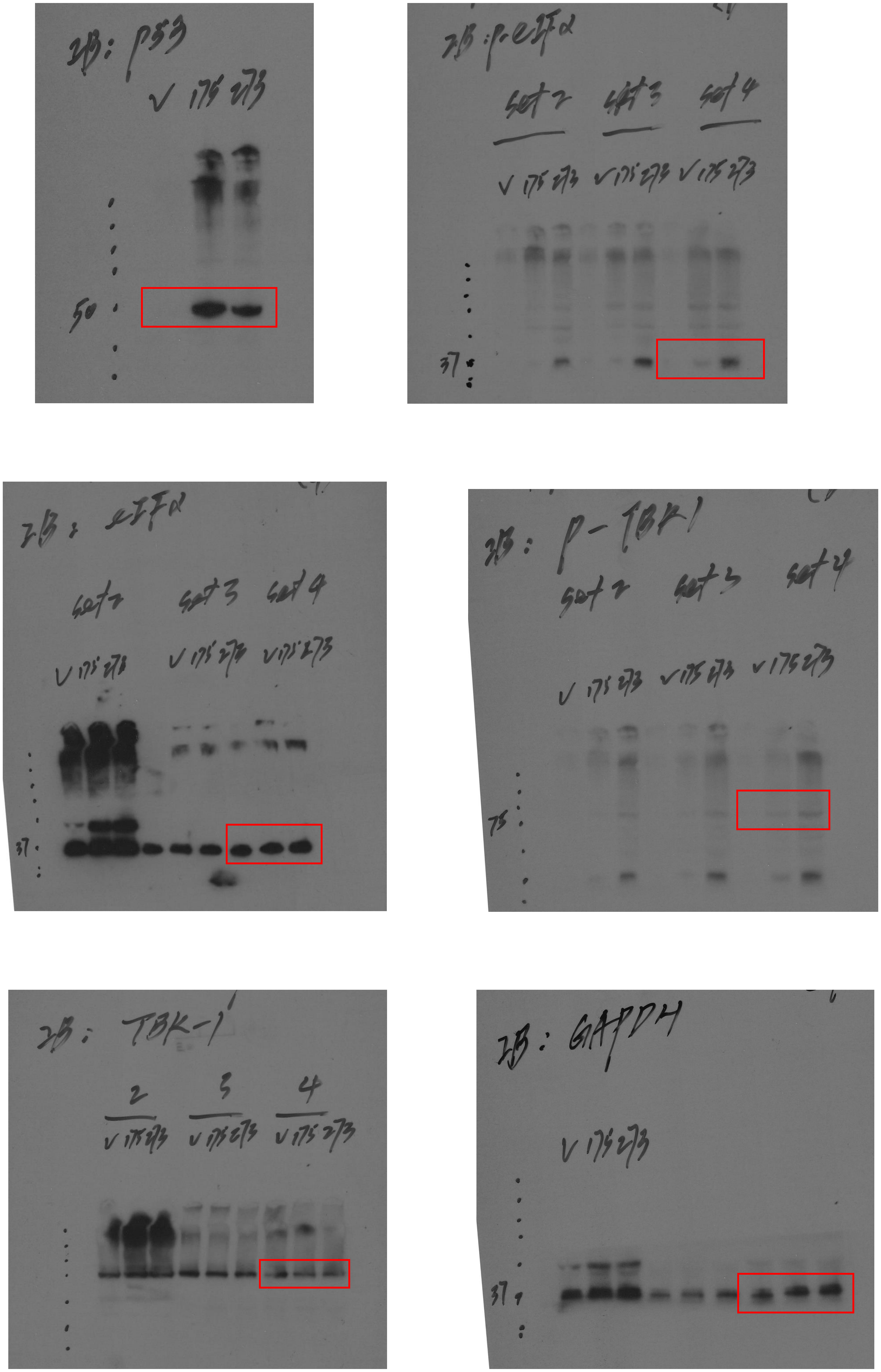
IB: p53
V 175 273
50
IB: p-eIF2α
set 2 set 3 set 4
V 175 273 V 175 273 V 175 273
37
IB: eIF2α
set 2 set 3 set 4
V 175 273 V 175 273 V 175 273
37
IB: p-TBK1
set 2 set 3 set 4
V 175 273 V 175 273 V 175 273
75
IB: TBK-1
2 3 4
V 175 273 V 175 273 V 175 273
IB: GAPDH
V 175 273
37

Fig. 8F

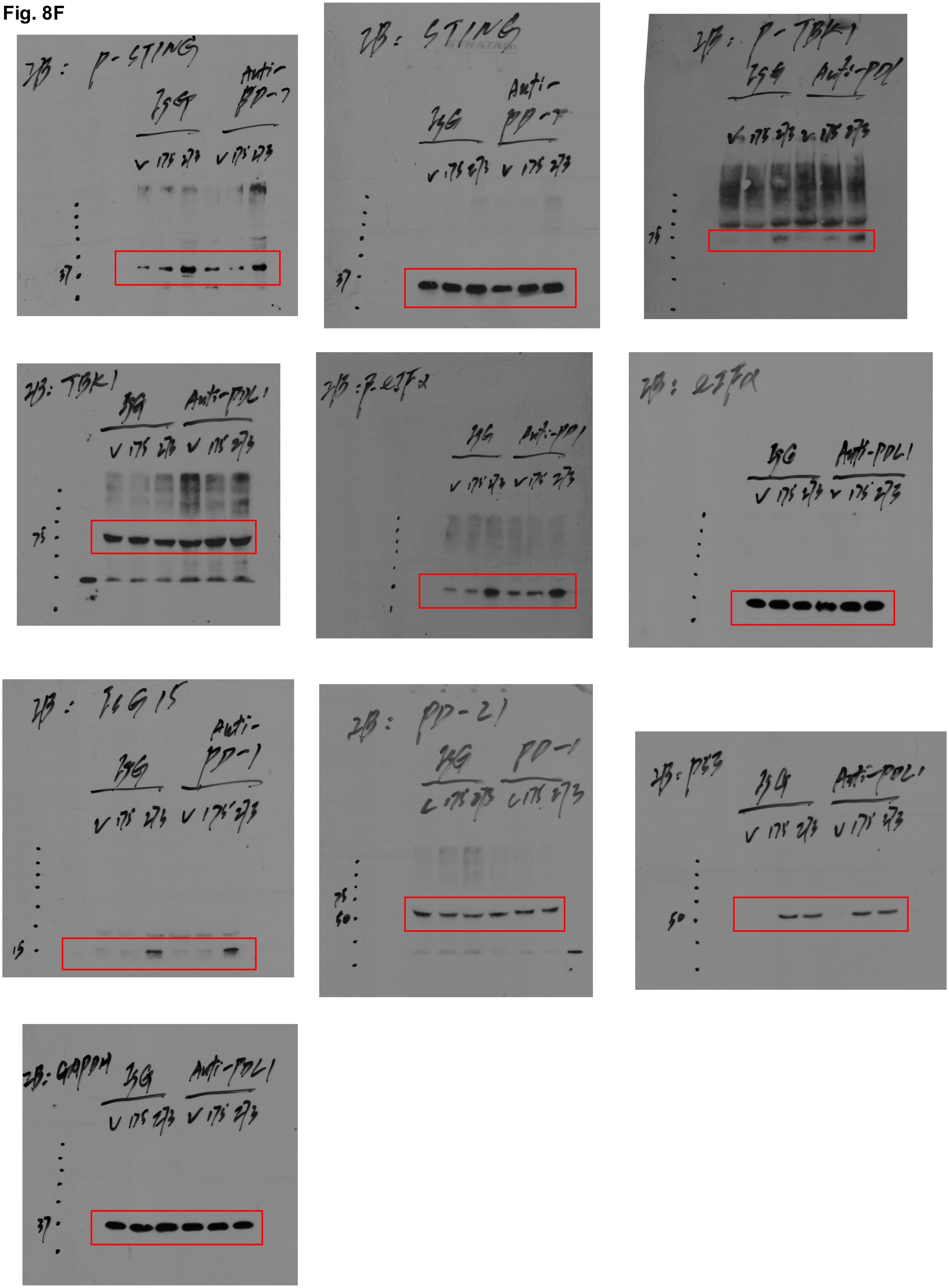

Fig. 9D

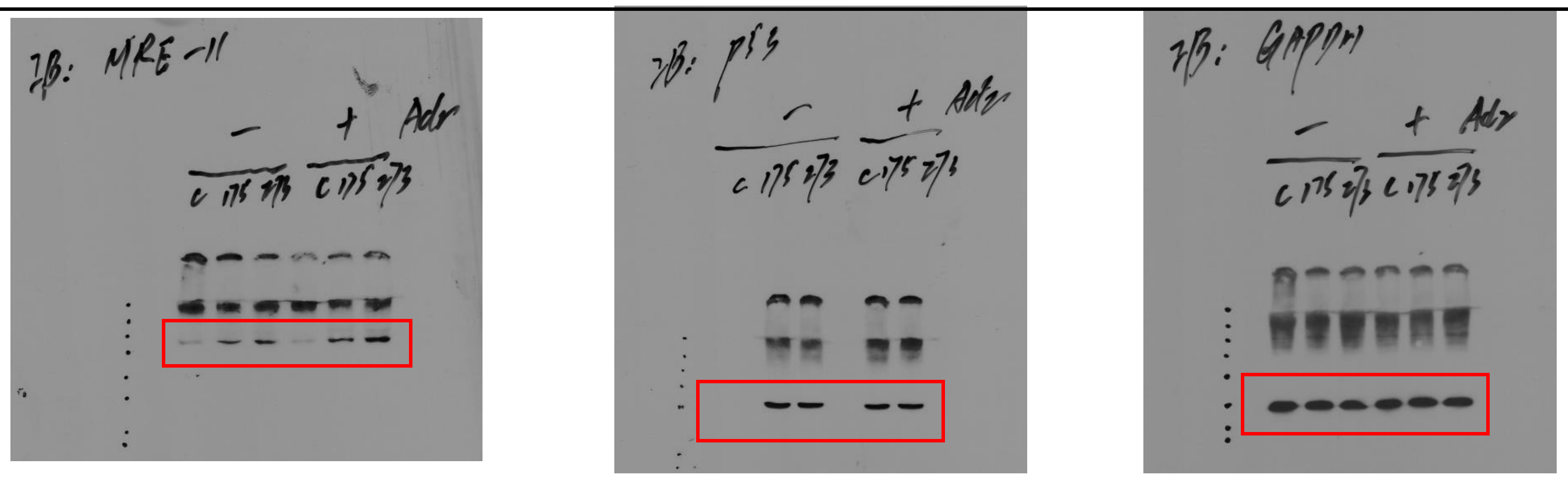

Fig.9E

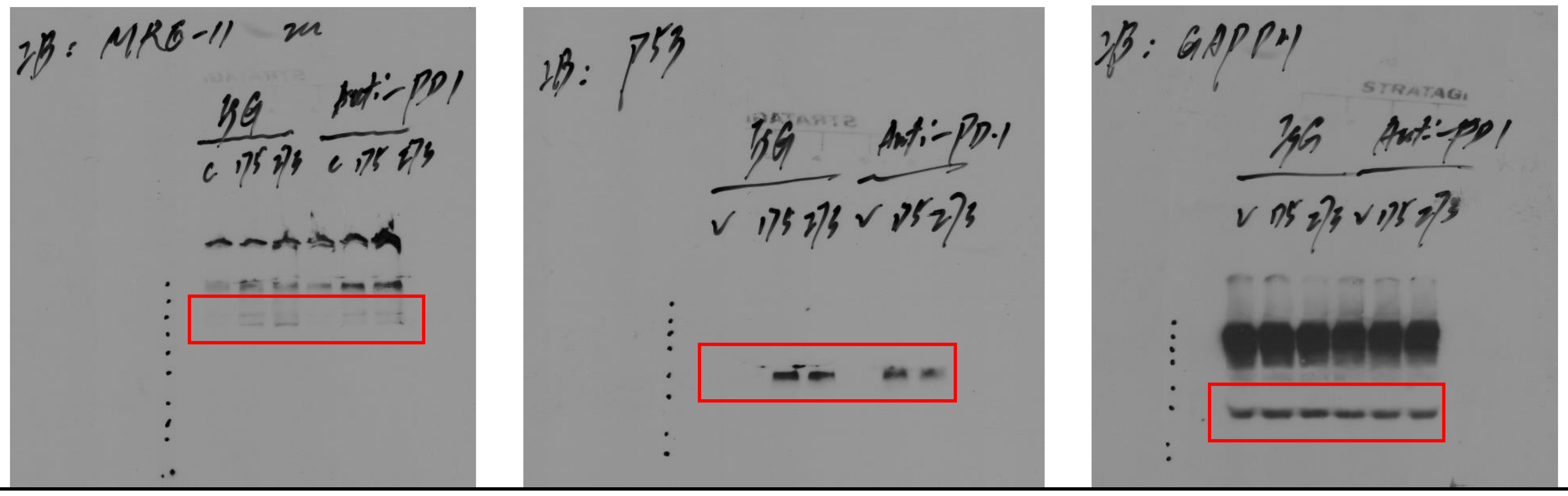

Fig.9G

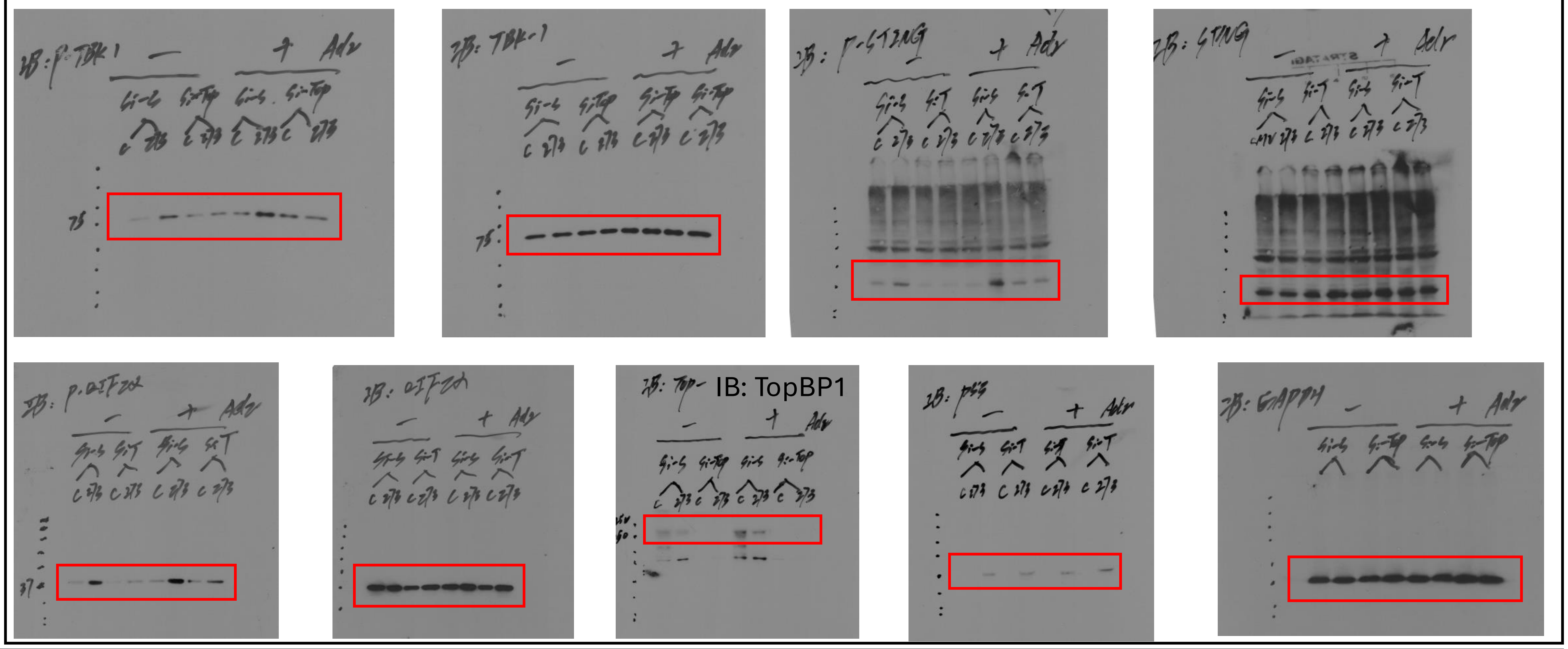

Fig.9I

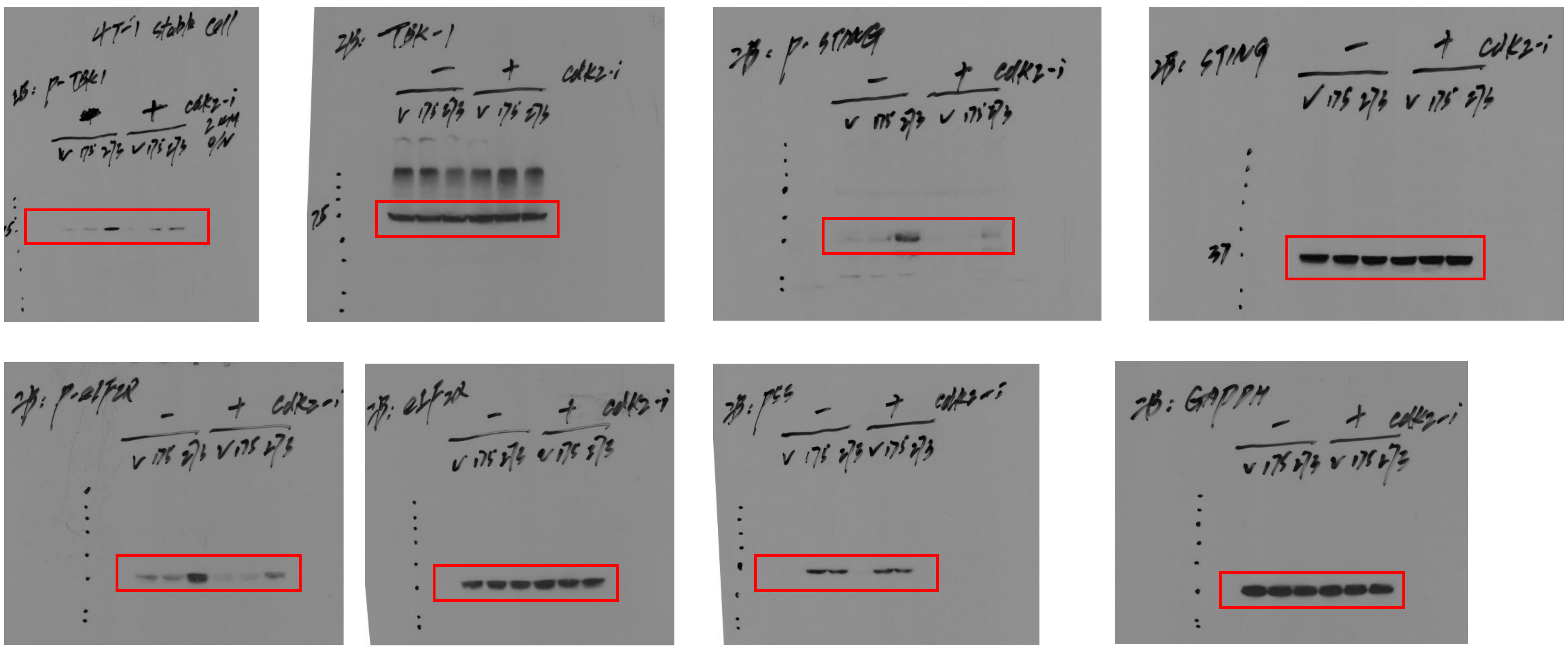

Fig. 9J

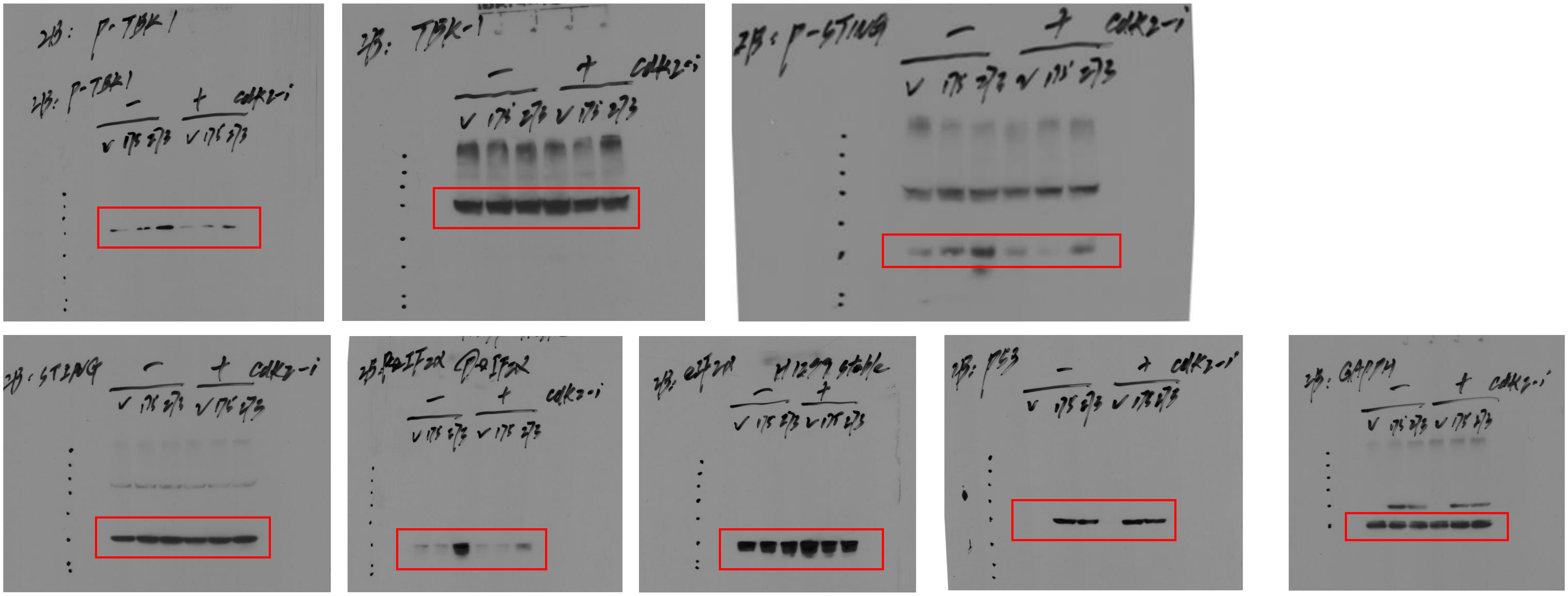

Fig. 10E

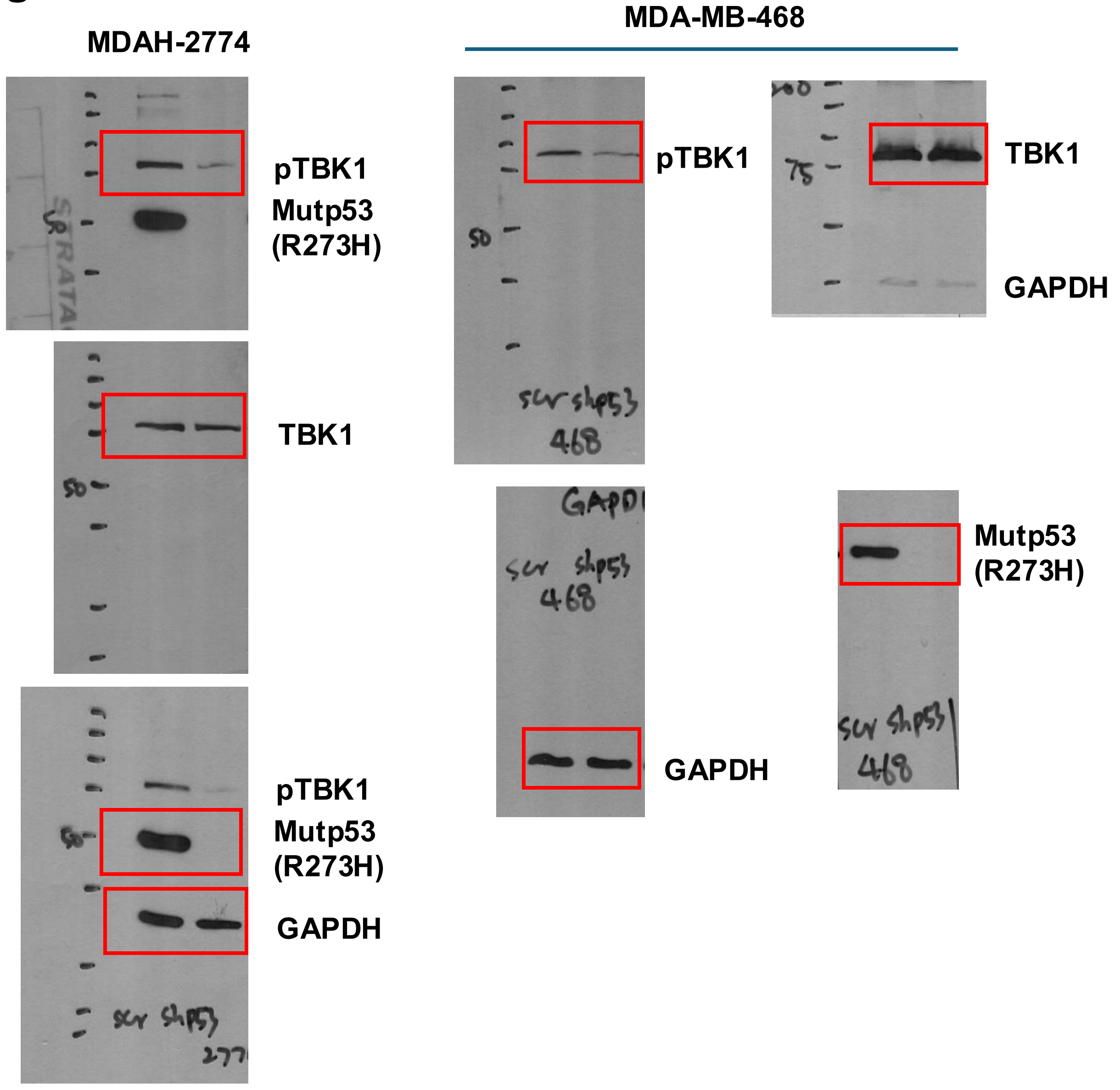

Fig. S4B

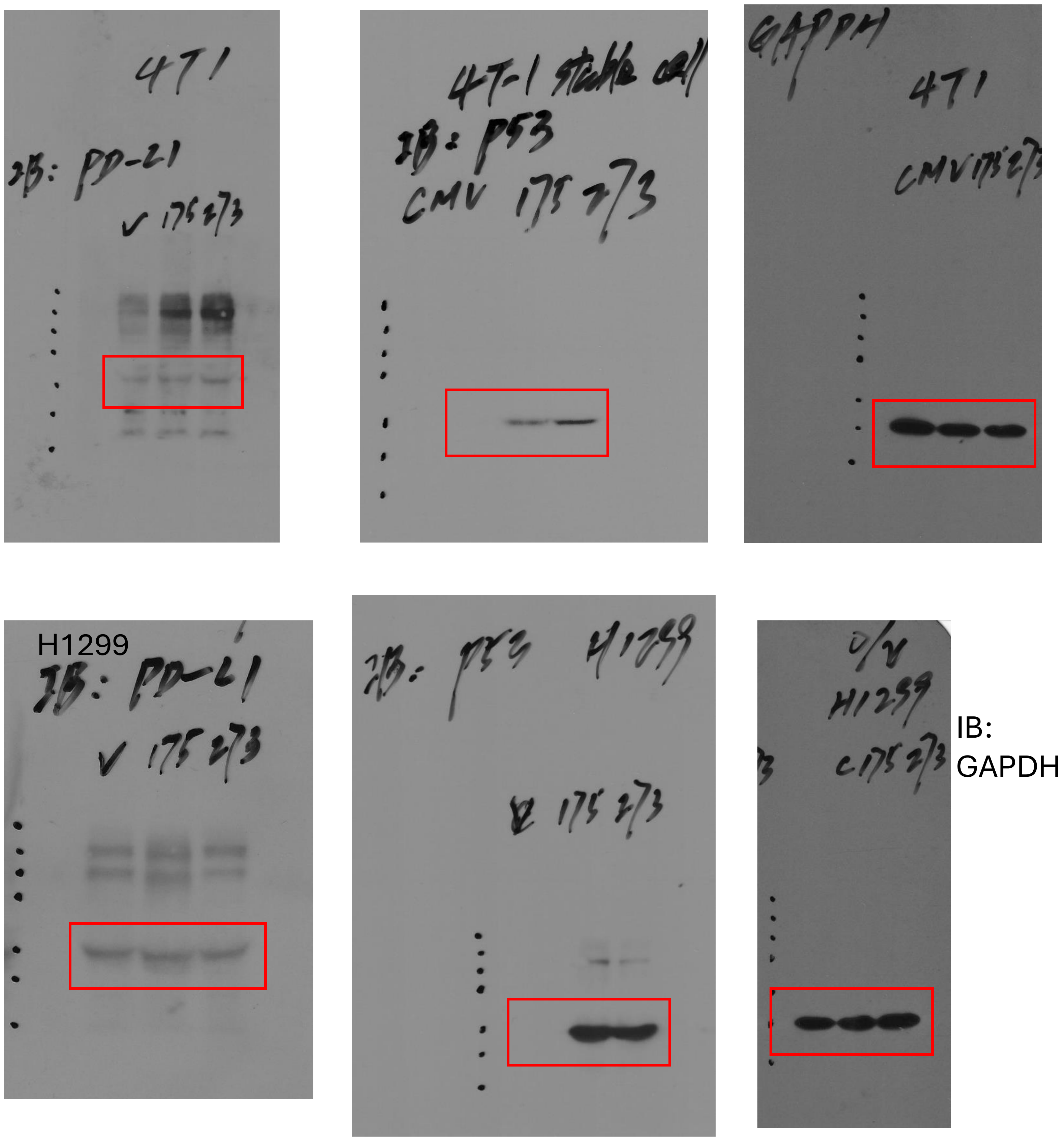

Supplement: Supplementary file 4 — Supplementary Data 1 [file 42003_2025_9050_MOESM4_ESM.pdf]
